# Supplementary material for: The Crk4-Cyc4 complex regulates G2/M transition in Toxoplasma gondii
Source: EMBO J. 2024 Apr 10;43(11):2094–126. doi: 10.1038/s44318-024-00095-4 (PMC11148040; doi:10.1038/s44318-024-00095-4)
Supplement: Supplementary file 2 — Dataset EV2 [file 44318_2024_95_MOESM2_ESM.zip › Dataset EV2/readme.docx]

**Dataset EV2. Entries used in phylogenetic analyses.**

Spreadsheet 1: Entries used in TgCrk4 phylogenetic analysis.

Spreadsheet 2: Entries used in TgiRD1phylogenetic analysis.
