# Supplementary material for: The Crk4-Cyc4 complex regulates G2/M transition in Toxoplasma gondii
Source: EMBO J. 2024 Apr 10;43(11):2094–126. doi: 10.1038/s44318-024-00095-4 (PMC11148040; doi:10.1038/s44318-024-00095-4)
Supplement: Supplementary file 3 — Dataset EV3 [file 44318_2024_95_MOESM3_ESM.zip › Dataset EV3/readme.docx]

**Dataset EV3. Proteomics data (IP/MS)**

Spreadsheet 1: Results of the short motif search

Spreadsheet 2: Results of the extended motif search

Spreadsheet 3: Essential genes containing short motif

Spreadsheet 4: Essential genes containing extended motif

Spreadsheet 5: Essential Crk4 substrates with short and extended motifs
