# Supplementary material for: The Crk4-Cyc4 complex regulates G2/M transition in Toxoplasma gondii
Source: EMBO J. 2024 Apr 10;43(11):2094–126. doi: 10.1038/s44318-024-00095-4 (PMC11148040; doi:10.1038/s44318-024-00095-4)
Supplement: Supplementary file 4 — Dataset EV4 [file 44318_2024_95_MOESM4_ESM.zip › Dataset EV4/readme.docx]

**Dataset EV4. Raw counts used in the expanded view figures.**

Spreadsheet 1: Raw counts for Fig. EV2 A

Spreadsheet 2: Raw counts for Fig. EV2 B

Spreadsheet 3: Raw counts for Fig. EV2 F
