# Supplementary material for: The Crk4-Cyc4 complex regulates G2/M transition in Toxoplasma gondii
Source: EMBO J. 2024 Apr 10;43(11):2094–126. doi: 10.1038/s44318-024-00095-4 (PMC11148040; doi:10.1038/s44318-024-00095-4)
Supplement: Supplementary file 5 — Dataset EV5 [file 44318_2024_95_MOESM5_ESM.zip › Dataset EV5/readme.docx]

**Dataset EV5. MaxQuant analysis of TgCrk4 global proteome and phosphoproteome**

Spreadsheet 1: Global proteome analysis

Spreadsheet 2: Global phosphoproteome analysis
