# Supplementary material for: The Crk4-Cyc4 complex regulates G2/M transition in Toxoplasma gondii
Source: EMBO J. 2024 Apr 10;43(11):2094–126. doi: 10.1038/s44318-024-00095-4 (PMC11148040; doi:10.1038/s44318-024-00095-4)
Supplement: Supplementary file 6 — Dataset EV6 [file 44318_2024_95_MOESM6_ESM.zip › Dataset EV6/readme.docx]

**Dataset EV6. Data analysis of the TgCrk4 global proteome.**

Spreadsheet 1: Differential classes by changes in expression

Spreadsheet 2: GO term upregulated entries.

Spreadsheet 3: GO term downregulated entries.
