# Supplementary material for: The Crk4-Cyc4 complex regulates G2/M transition in Toxoplasma gondii
Source: EMBO J. 2024 Apr 10;43(11):2094–126. doi: 10.1038/s44318-024-00095-4 (PMC11148040; doi:10.1038/s44318-024-00095-4)
Supplement: Supplementary file 7 — Dataset EV7 [file 44318_2024_95_MOESM7_ESM.zip › Dataset EV7/readme.docx]

**Dataset EV7. Analysis of TgCrk4 global phosphoproteome.**

Spreadsheet 1: Differential classes by changes in expression

Spreadsheet 2: GO term downregulated entries.

Spreadsheet 3: Differential classes for heatmap.
