# Supplementary material for: The Crk4-Cyc4 complex regulates G2/M transition in Toxoplasma gondii
Source: EMBO J. 2024 Apr 10;43(11):2094–126. doi: 10.1038/s44318-024-00095-4 (PMC11148040; doi:10.1038/s44318-024-00095-4)
Supplement: Supplementary file 8 — Dataset EV8 [file 44318_2024_95_MOESM8_ESM.zip › Dataset EV8/readme.docx]

**Dataset EV8. Search for G_2_-dependent phosphorylation motifs and TgCrk4 substrates.**

Spreadsheet 1: Downregulated phosphorylation motifs after 30 min TgCrk4 deficiency

Spreadsheet 2: Downregulated phosphorylation motifs after 4 hours TgCrk4 deficiency

Spreadsheet 3: Upregulated phosphorylation motifs after 30 min TgCrk4 deficiency

Spreadsheet 4: Upregulated phosphorylation motifs after 4 hours TgCrk4 deficiency

Spreadsheet 5: Putative TgCrk4 substrates with short proline-driven motif

Spreadsheet 6: Putative TgCrk4 substrates with extended proline-driven motif
