# Supplementary figures and images for: The Crk4-Cyc4 complex regulates G2/M transition in Toxoplasma gondii (part 1 of 2)
Source: EMBO J. 2024 Apr 10;43(11):2094–126. doi: 10.1038/s44318-024-00095-4 (PMC11148040; doi:10.1038/s44318-024-00095-4)

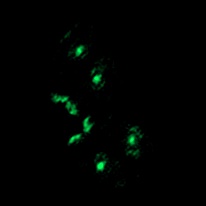

Supplement: Supplementary file 9 — Source data Fig. 1 [file 44318_2024_95_MOESM9_ESM.zip › SD Figure 1/1H/MORN1 Cyc4 mitosis-1.jpg]

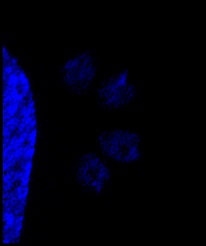

Supplement: Supplementary file 9 — Source data Fig. 1 [file 44318_2024_95_MOESM9_ESM.zip › SD Figure 1/1H/DAPI Cyc4 mitosis-2.jpg]

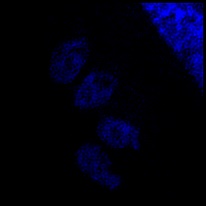

Supplement: Supplementary file 9 — Source data Fig. 1 [file 44318_2024_95_MOESM9_ESM.zip › SD Figure 1/1H/DAPI Cyc4 mitosis-1.jpg]

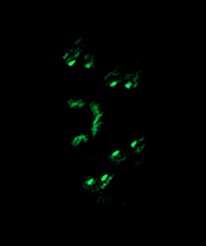

Supplement: Supplementary file 9 — Source data Fig. 1 [file 44318_2024_95_MOESM9_ESM.zip › SD Figure 1/1H/MORN1 Cyc4 mitosis-2.jpg]

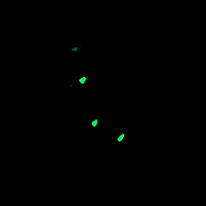

Supplement: Supplementary file 9 — Source data Fig. 1 [file 44318_2024_95_MOESM9_ESM.zip › SD Figure 1/1H/centrin Cyc4 G1.jpg]

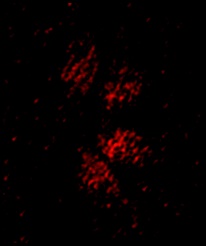

Supplement: Supplementary file 9 — Source data Fig. 1 [file 44318_2024_95_MOESM9_ESM.zip › SD Figure 1/1H/HA Cyc4 mitosis-2.jpg]

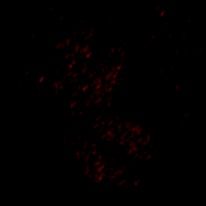

Supplement: Supplementary file 9 — Source data Fig. 1 [file 44318_2024_95_MOESM9_ESM.zip › SD Figure 1/1H/HA Cyc4 mitosis-1.jpg]

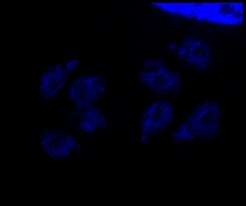

Supplement: Supplementary file 9 — Source data Fig. 1 [file 44318_2024_95_MOESM9_ESM.zip › SD Figure 1/1H/DAPI Cyc4 S-phase.jpg]

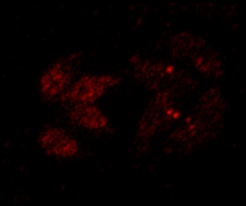

Supplement: Supplementary file 9 — Source data Fig. 1 [file 44318_2024_95_MOESM9_ESM.zip › SD Figure 1/1H/HA Cyc4 S-phase.jpg]

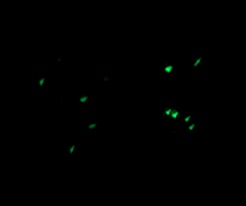

Supplement: Supplementary file 9 — Source data Fig. 1 [file 44318_2024_95_MOESM9_ESM.zip › SD Figure 1/1H/centrin Cyc4 S-phase.jpg]

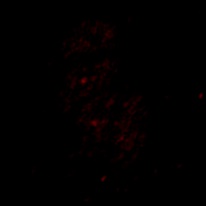

Supplement: Supplementary file 9 — Source data Fig. 1 [file 44318_2024_95_MOESM9_ESM.zip › SD Figure 1/1H/HA Cyc4 G1.jpg]

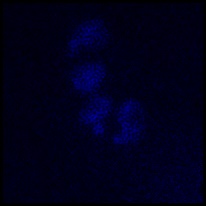

Supplement: Supplementary file 9 — Source data Fig. 1 [file 44318_2024_95_MOESM9_ESM.zip › SD Figure 1/1H/DAPI Cyc4 G1.jpg]

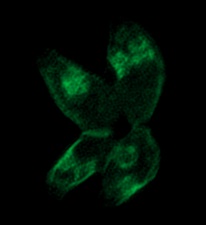

Supplement: Supplementary file 9 — Source data Fig. 1 [file 44318_2024_95_MOESM9_ESM.zip › SD Figure 1/1H/IMC1 Cyc4 budding.jpg]

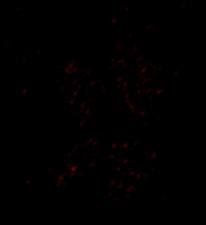

Supplement: Supplementary file 9 — Source data Fig. 1 [file 44318_2024_95_MOESM9_ESM.zip › SD Figure 1/1H/HA Cyc4 budding.jpg]

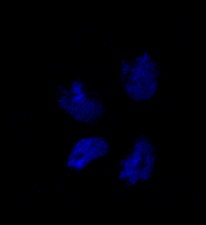

Supplement: Supplementary file 9 — Source data Fig. 1 [file 44318_2024_95_MOESM9_ESM.zip › SD Figure 1/1H/DAPI Cyc4 budding.jpg]

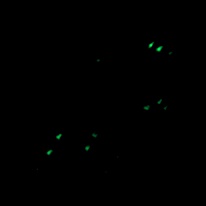

Supplement: Supplementary file 9 — Source data Fig. 1 [file 44318_2024_95_MOESM9_ESM.zip › SD Figure 1/1F/centrin Crk4 G1.jpg]

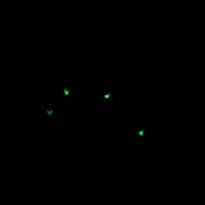

Supplement: Supplementary file 9 — Source data Fig. 1 [file 44318_2024_95_MOESM9_ESM.zip › SD Figure 1/1F/centrin Crk4 S-phase.jpg]

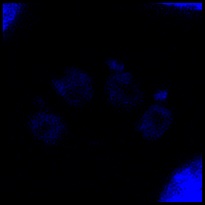

Supplement: Supplementary file 9 — Source data Fig. 1 [file 44318_2024_95_MOESM9_ESM.zip › SD Figure 1/1F/DAPI Crk4 S-phase.jpg]

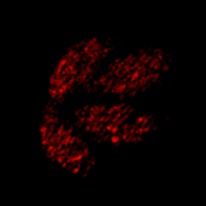

Supplement: Supplementary file 9 — Source data Fig. 1 [file 44318_2024_95_MOESM9_ESM.zip › SD Figure 1/1F/myc Crk4 budding.jpg]

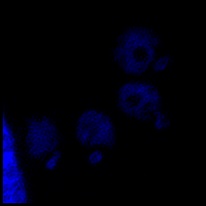

Supplement: Supplementary file 9 — Source data Fig. 1 [file 44318_2024_95_MOESM9_ESM.zip › SD Figure 1/1F/DAPI Crk4 G1.jpg]

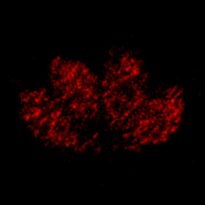

Supplement: Supplementary file 9 — Source data Fig. 1 [file 44318_2024_95_MOESM9_ESM.zip › SD Figure 1/1F/myc Crk4 S-phase.jpg]

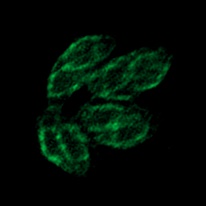

Supplement: Supplementary file 9 — Source data Fig. 1 [file 44318_2024_95_MOESM9_ESM.zip › SD Figure 1/1F/IMC1 Crk4 budding.jpg]

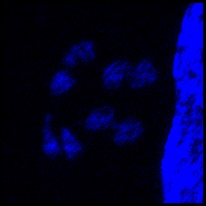

Supplement: Supplementary file 9 — Source data Fig. 1 [file 44318_2024_95_MOESM9_ESM.zip › SD Figure 1/1F/DAPI Crk4 budding.jpg]

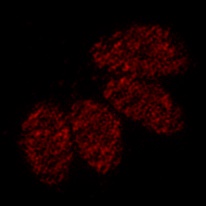

Supplement: Supplementary file 9 — Source data Fig. 1 [file 44318_2024_95_MOESM9_ESM.zip › SD Figure 1/1F/myc Crk4 G1.jpg]

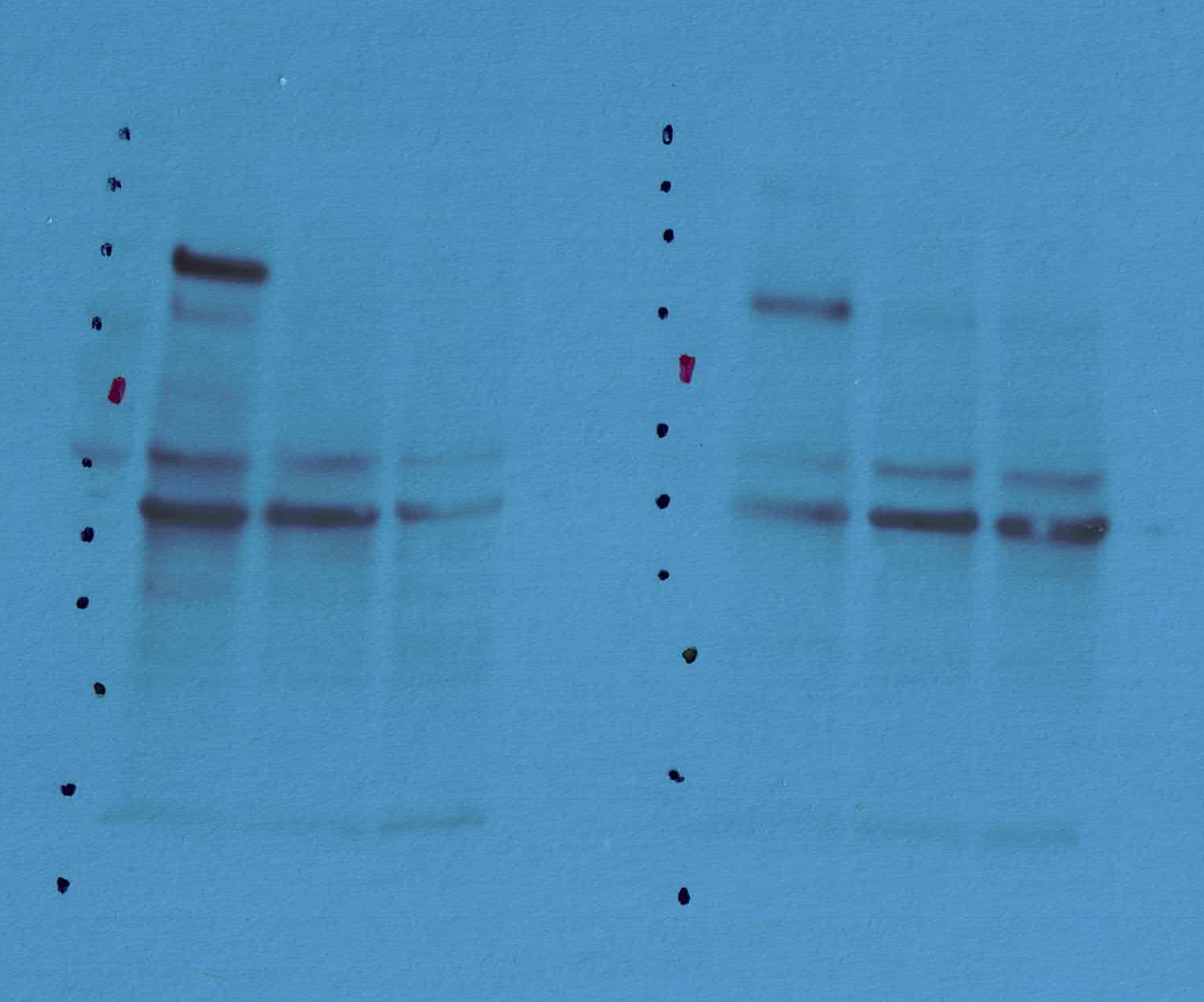

Supplement: Supplementary file 9 — Source data Fig. 1 [file 44318_2024_95_MOESM9_ESM.zip › SD Figure 1/1A/Crk4 HA.tif]

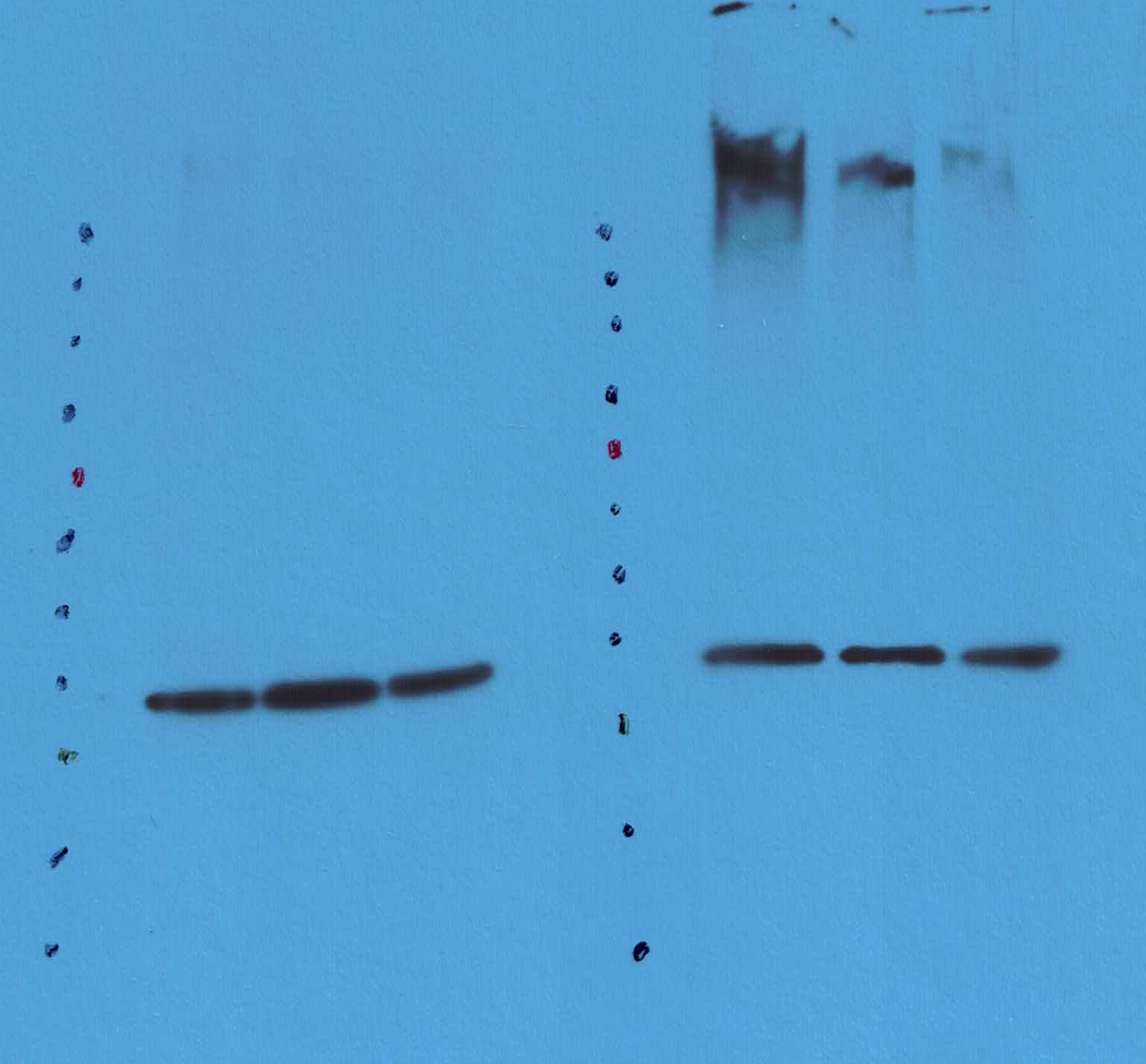

Supplement: Supplementary file 9 — Source data Fig. 1 [file 44318_2024_95_MOESM9_ESM.zip › SD Figure 1/1A/Crk4 GRA7.tif]

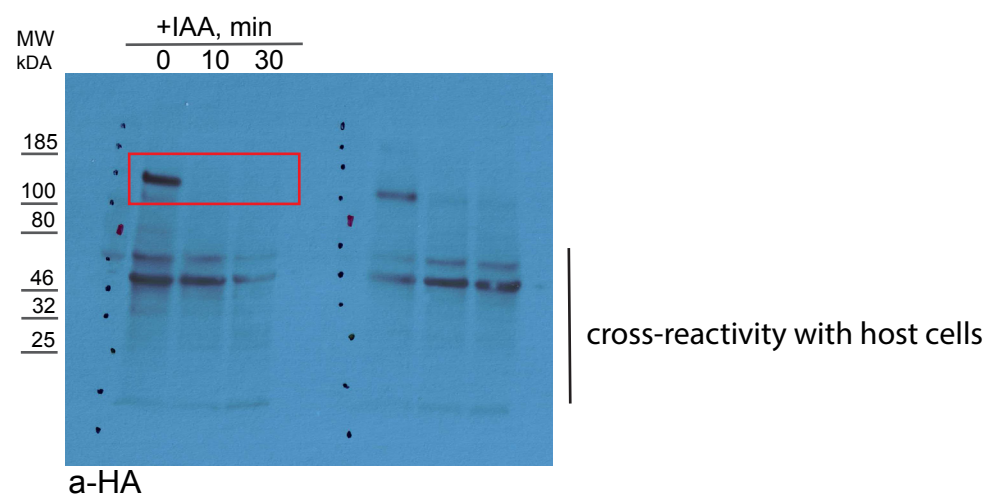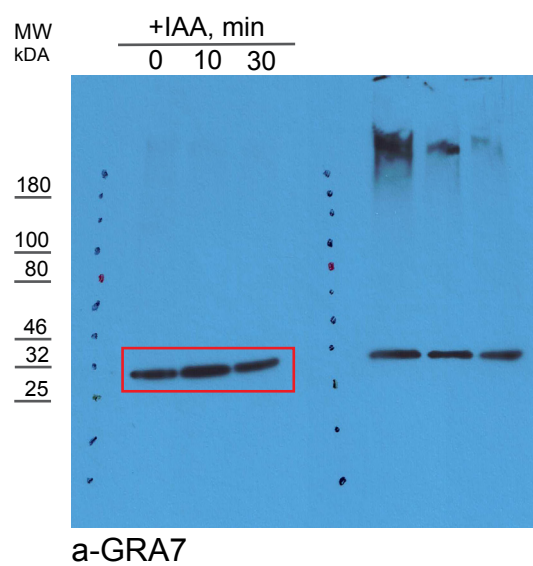

Supplement: Supplementary file 9 — Source data Fig. 1 [file 44318_2024_95_MOESM9_ESM.zip › SD Figure 1/1A/readme.pdf]

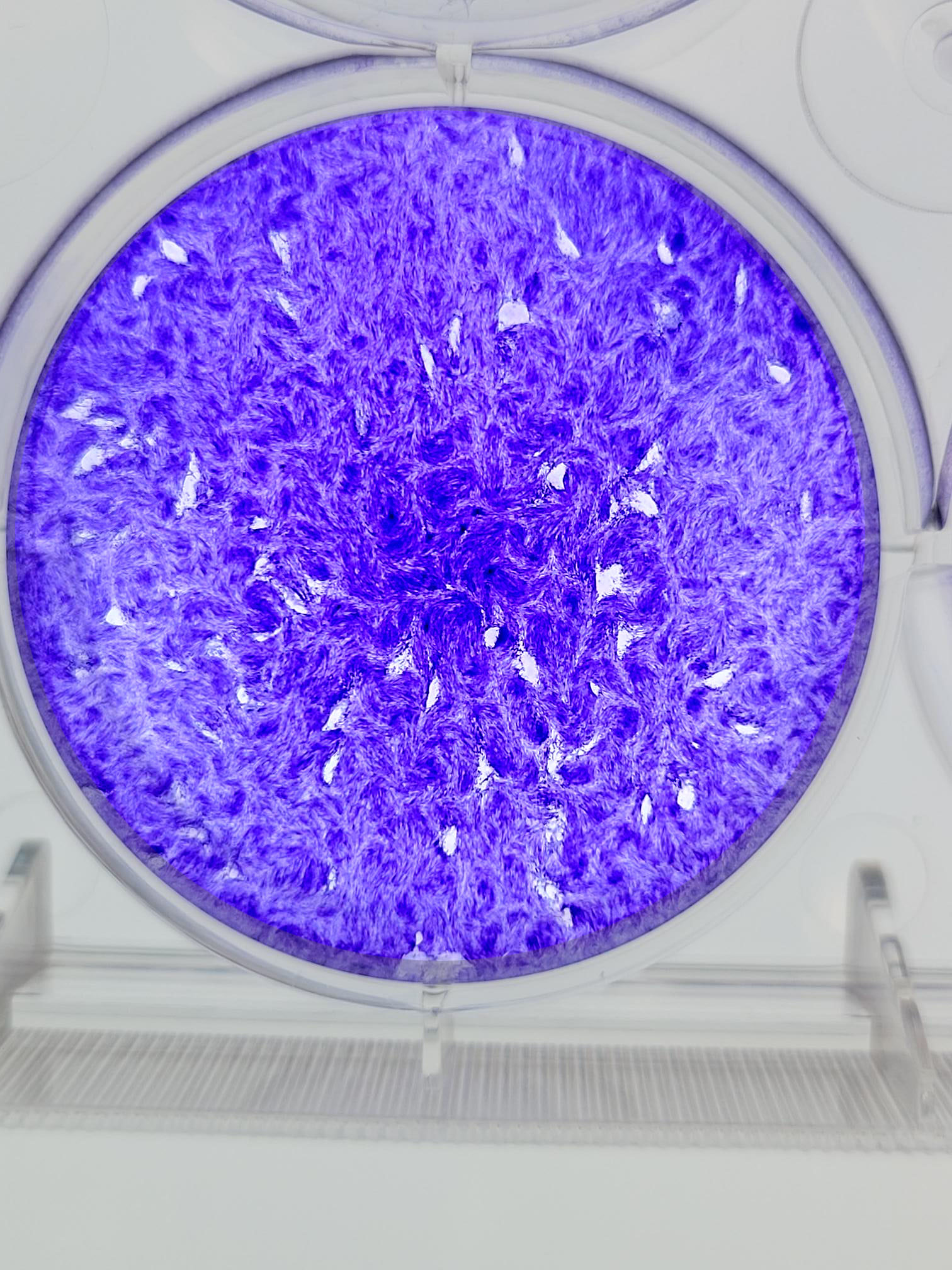

Supplement: Supplementary file 9 — Source data Fig. 1 [file 44318_2024_95_MOESM9_ESM.zip › SD Figure 1/1B/TgCrk4 -aux.jpg]

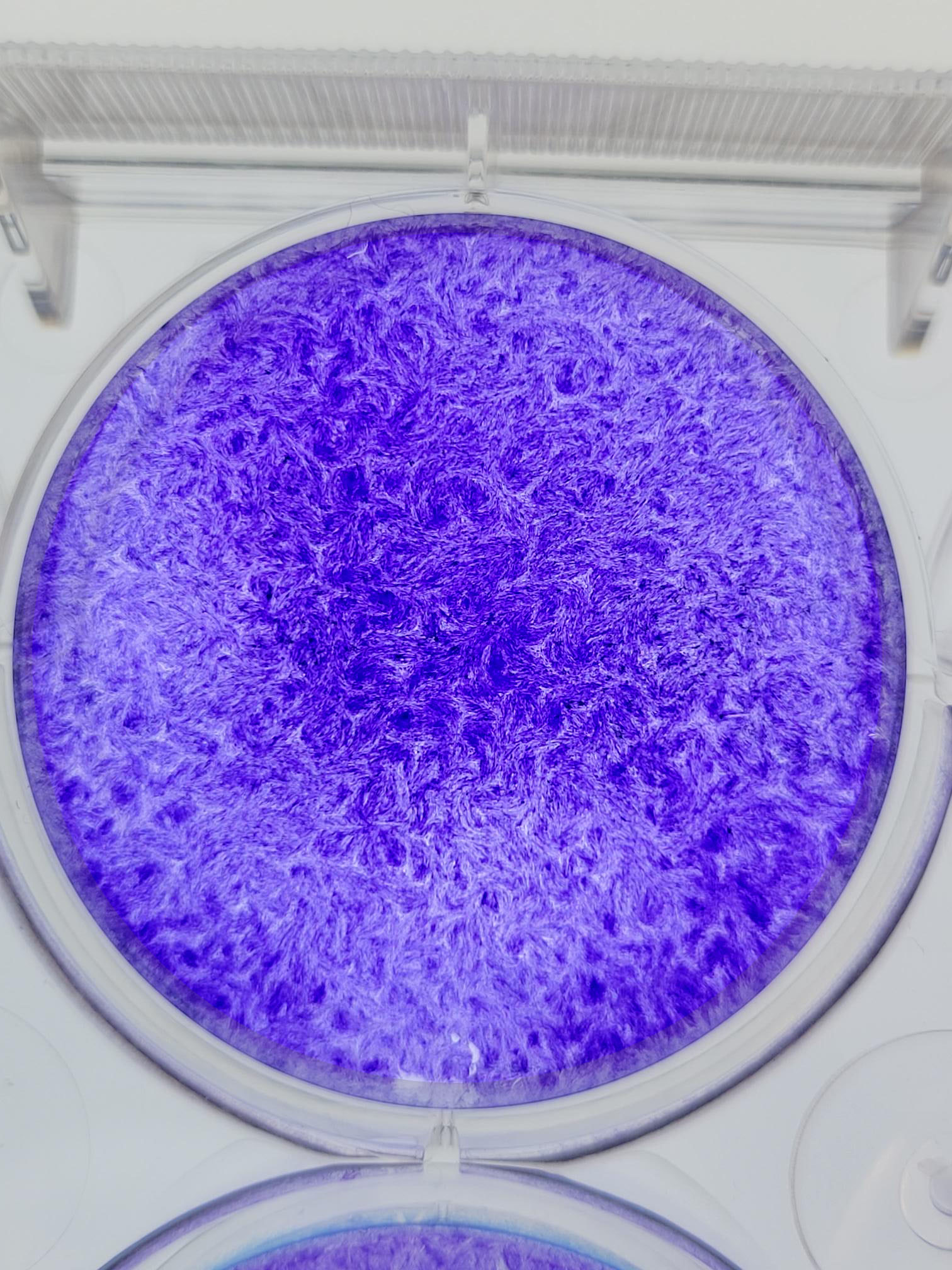

Supplement: Supplementary file 9 — Source data Fig. 1 [file 44318_2024_95_MOESM9_ESM.zip › SD Figure 1/1B/TgCrk4 +aux.jpg]

RH TgCrk4<sup>AID-HA</sup>

-IAA

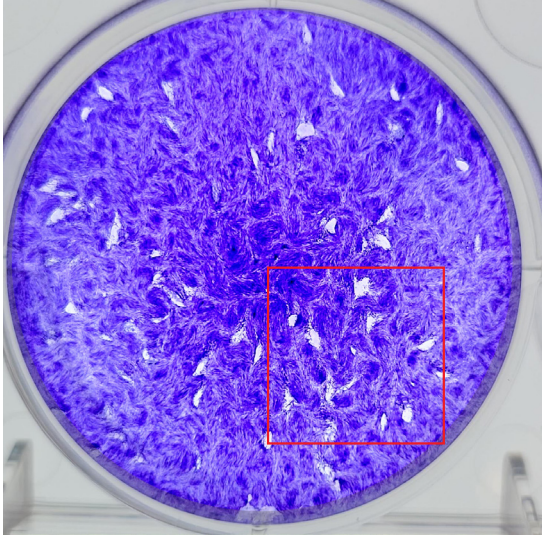

+IAA

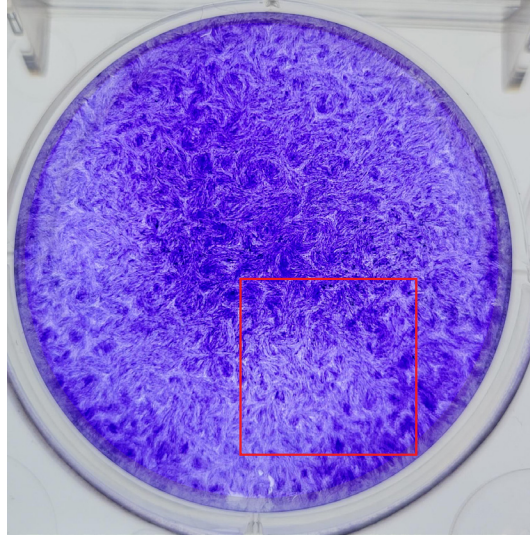

Supplement: Supplementary file 9 — Source data Fig. 1 [file 44318_2024_95_MOESM9_ESM.zip › SD Figure 1/1B/readme.pdf]

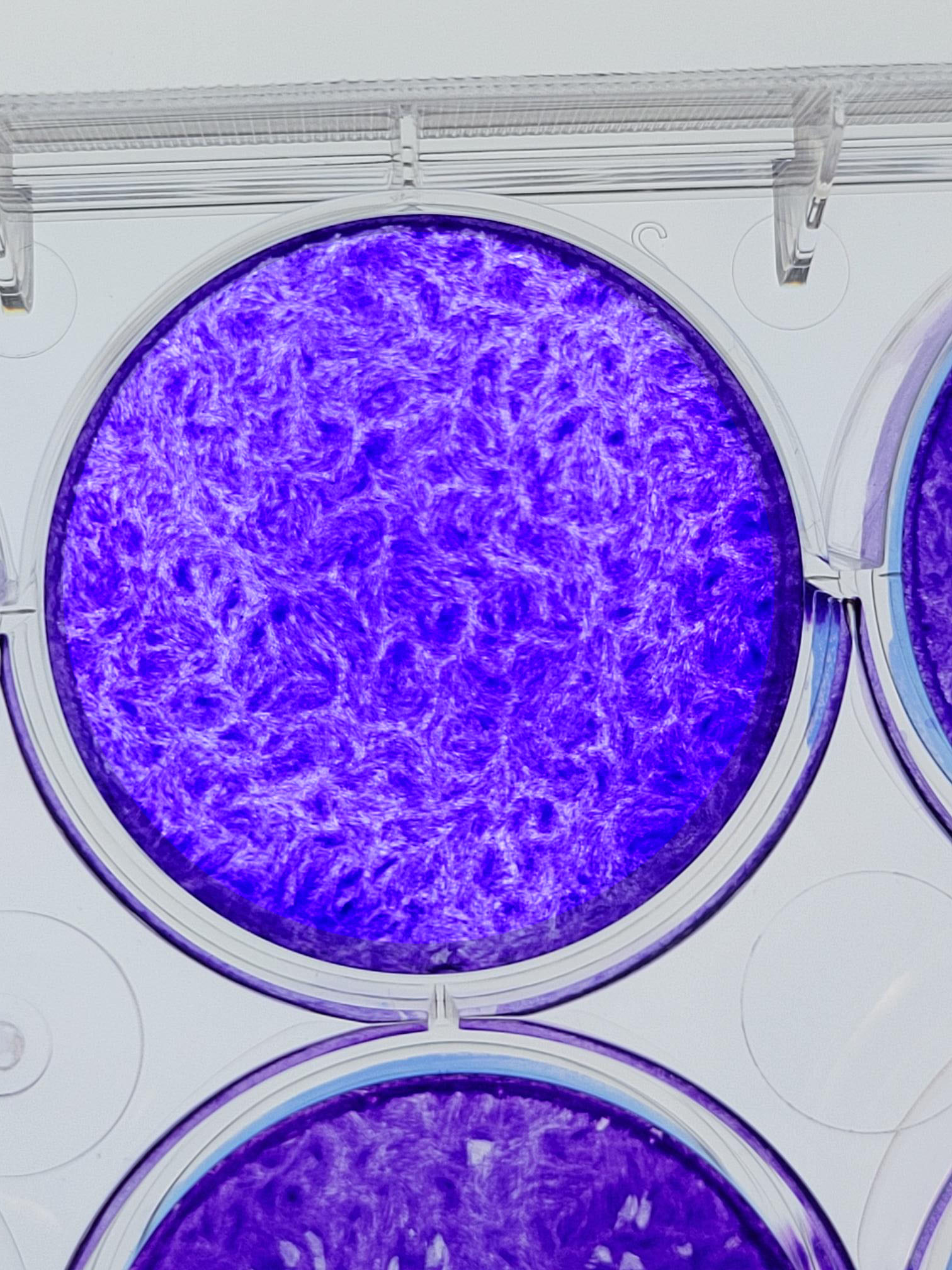

Supplement: Supplementary file 9 — Source data Fig. 1 [file 44318_2024_95_MOESM9_ESM.zip › SD Figure 1/1D/TgCyc4 Tati +ATc.jpg]

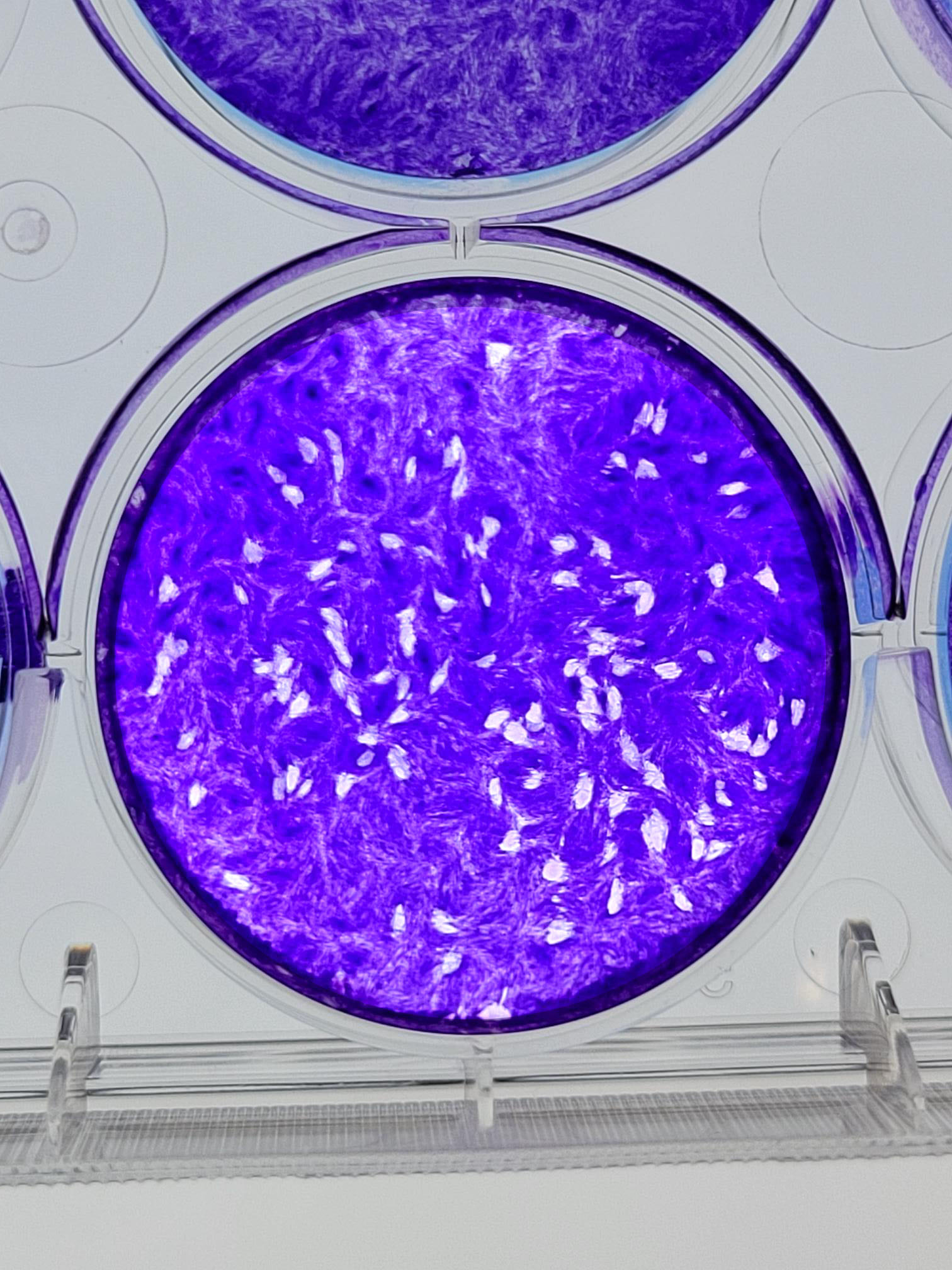

Supplement: Supplementary file 9 — Source data Fig. 1 [file 44318_2024_95_MOESM9_ESM.zip › SD Figure 1/1D/TgCyc4 Tati -ATc.jpg]

RH-Tati<sup>HATgCyc4</sup>

-ATc

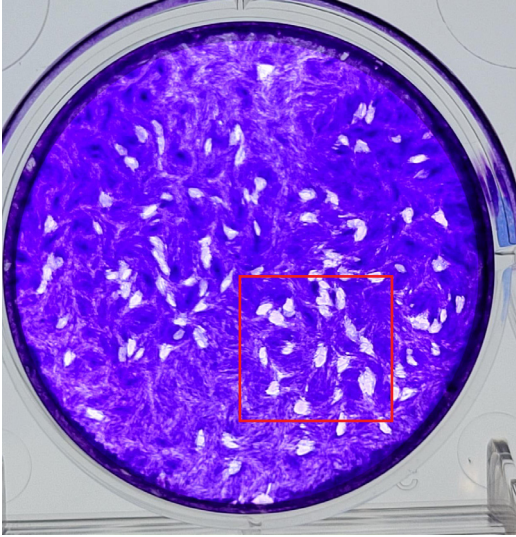

+ATc

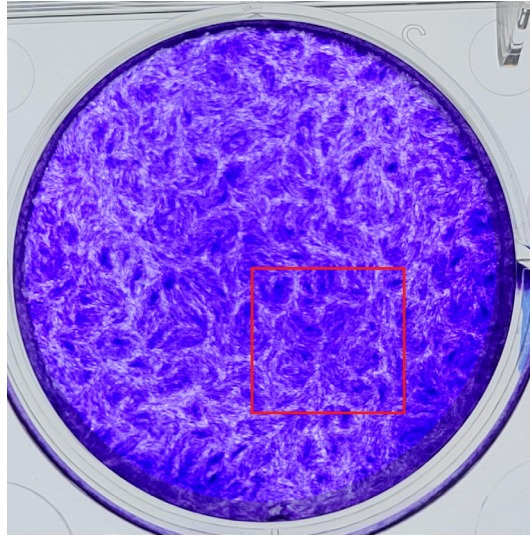

Supplement: Supplementary file 9 — Source data Fig. 1 [file 44318_2024_95_MOESM9_ESM.zip › SD Figure 1/1D/readme.pdf]

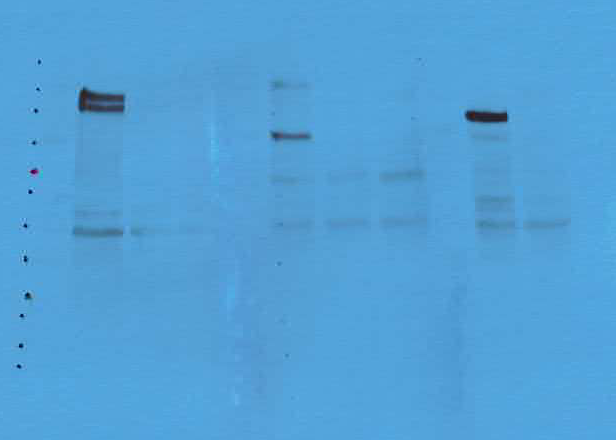

Supplement: Supplementary file 9 — Source data Fig. 1 [file 44318_2024_95_MOESM9_ESM.zip › SD Figure 1/1C/Cyc4 HA.tif]

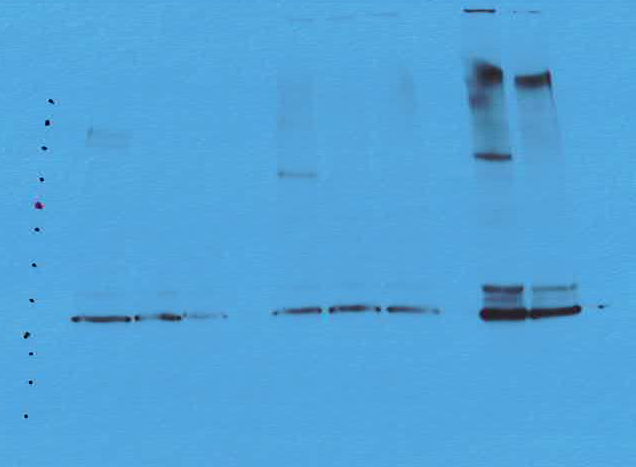

Supplement: Supplementary file 9 — Source data Fig. 1 [file 44318_2024_95_MOESM9_ESM.zip › SD Figure 1/1C/Cyc4 GRA7.tif]

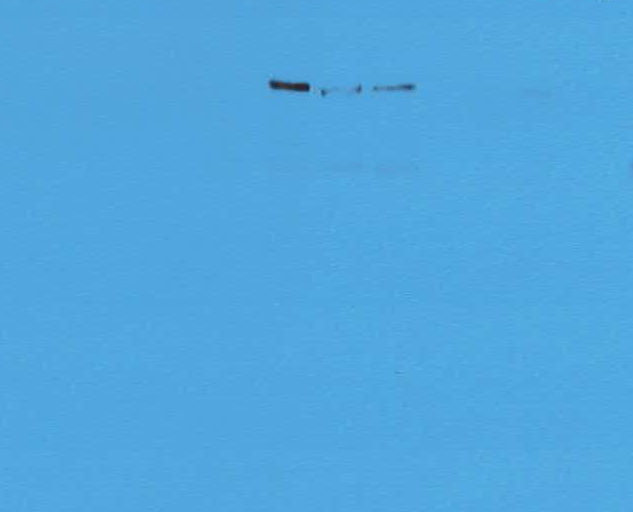

Supplement: Supplementary file 9 — Source data Fig. 1 [file 44318_2024_95_MOESM9_ESM.zip › SD Figure 1/1C/Cyc4 myc.tif]

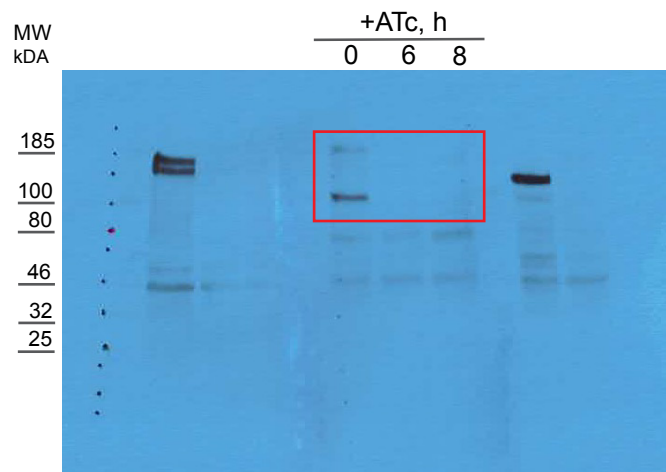

a-HA

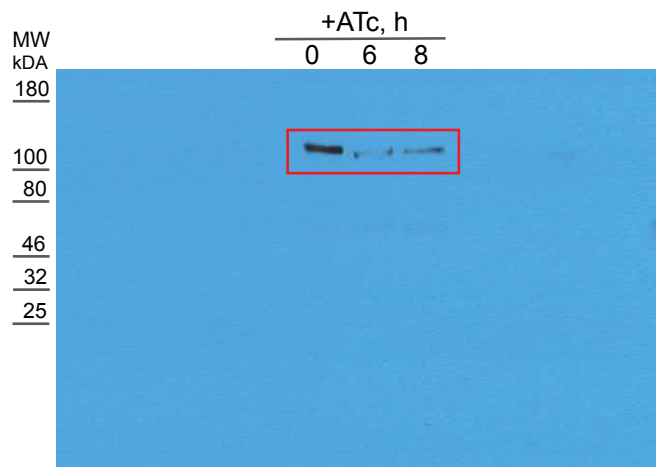

a-myc

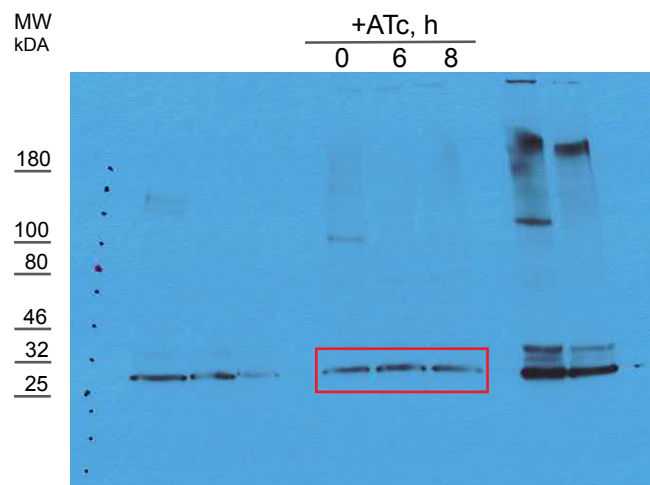

a-GRA7

Supplement: Supplementary file 9 — Source data Fig. 1 [file 44318_2024_95_MOESM9_ESM.zip › SD Figure 1/1C/readme.pdf]

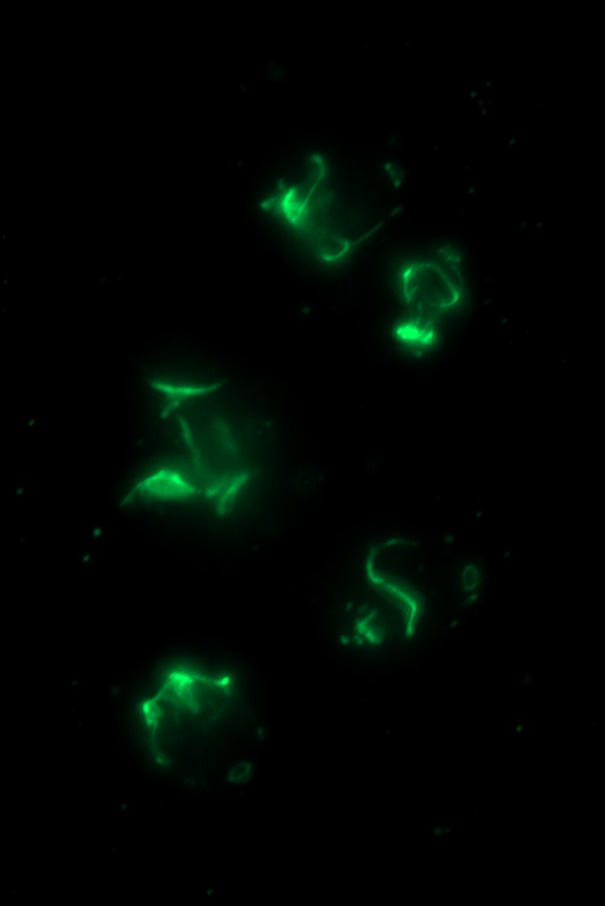

Supplement: Supplementary file 10 — Source data Fig. 2 [file 44318_2024_95_MOESM10_ESM.zip › SD Figure 2/2I/Crk4 +aux 4h MORN1.jpg]

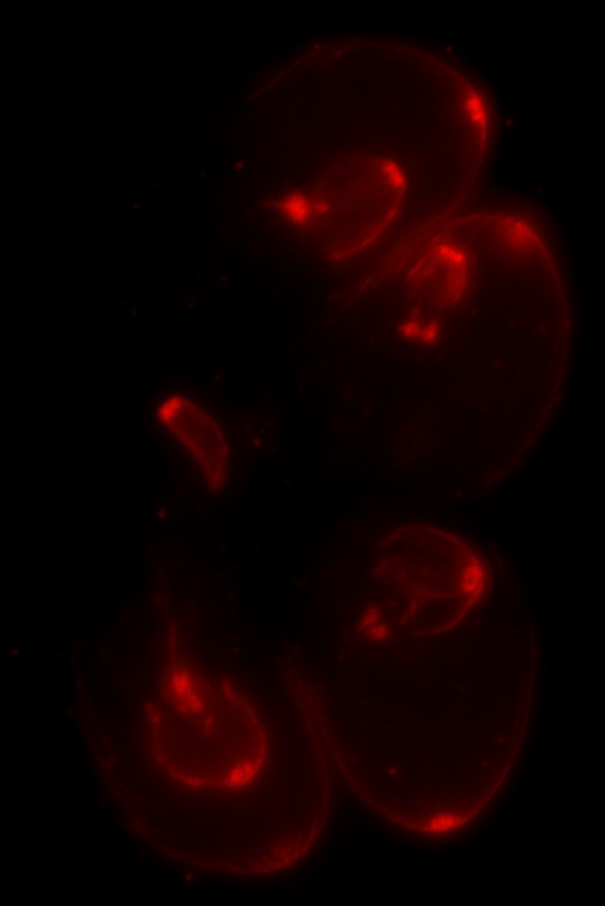

Supplement: Supplementary file 10 — Source data Fig. 2 [file 44318_2024_95_MOESM10_ESM.zip › SD Figure 2/2I/Crk4 +aux 4h Tubulin.jpg]

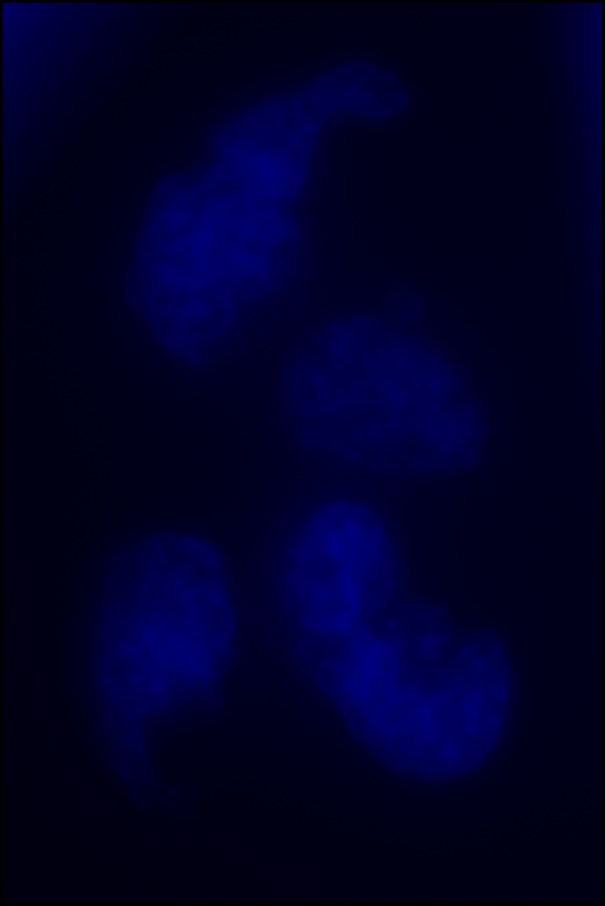

Supplement: Supplementary file 10 — Source data Fig. 2 [file 44318_2024_95_MOESM10_ESM.zip › SD Figure 2/2I/Crk4 +aux 4h DAPI.jpg]

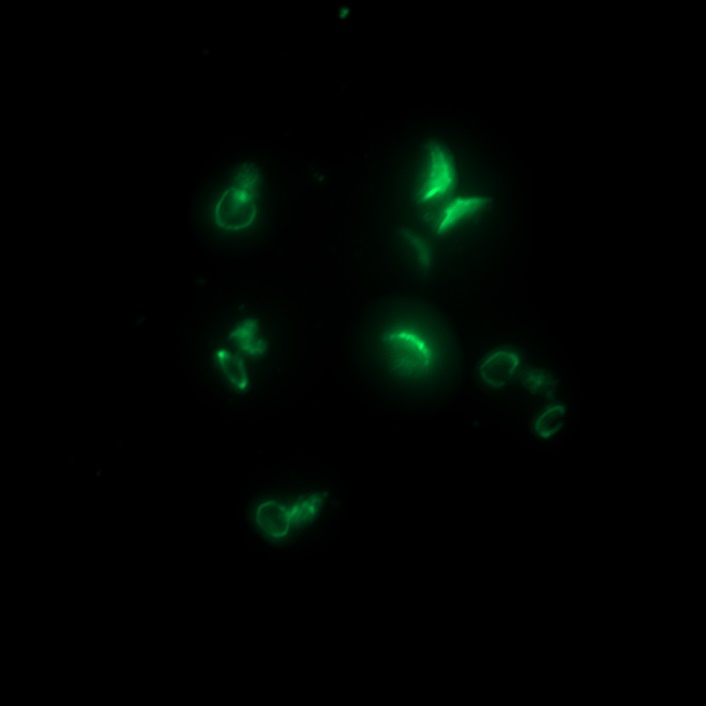

Supplement: Supplementary file 10 — Source data Fig. 2 [file 44318_2024_95_MOESM10_ESM.zip › SD Figure 2/2H/Crk4 +aux 4h MORN1.jpg]

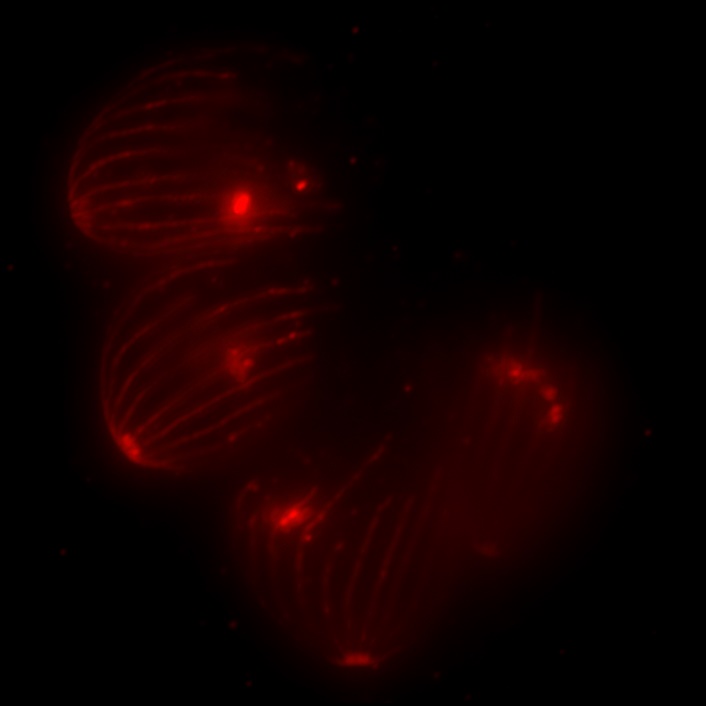

Supplement: Supplementary file 10 — Source data Fig. 2 [file 44318_2024_95_MOESM10_ESM.zip › SD Figure 2/2H/Crk4 +aux 4h Tubulin.jpg]

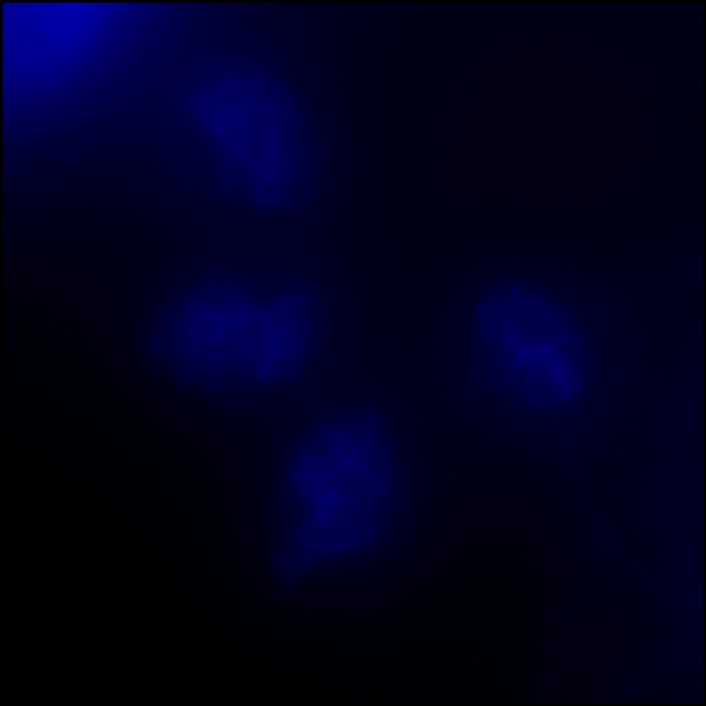

Supplement: Supplementary file 10 — Source data Fig. 2 [file 44318_2024_95_MOESM10_ESM.zip › SD Figure 2/2H/Crk4 +aux 4h DAPI.jpg]

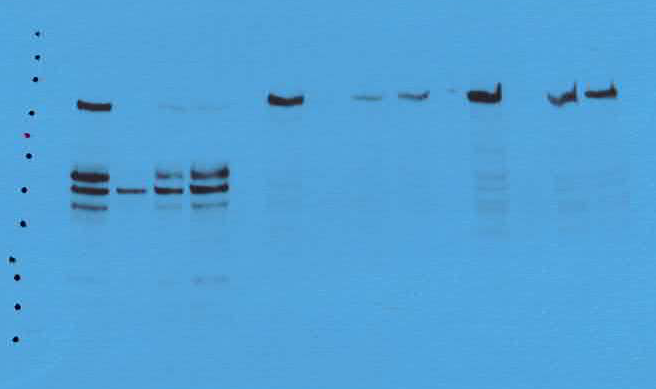

Supplement: Supplementary file 10 — Source data Fig. 2 [file 44318_2024_95_MOESM10_ESM.zip › SD Figure 2/2A/Crk4 Crk6 HA.tif]

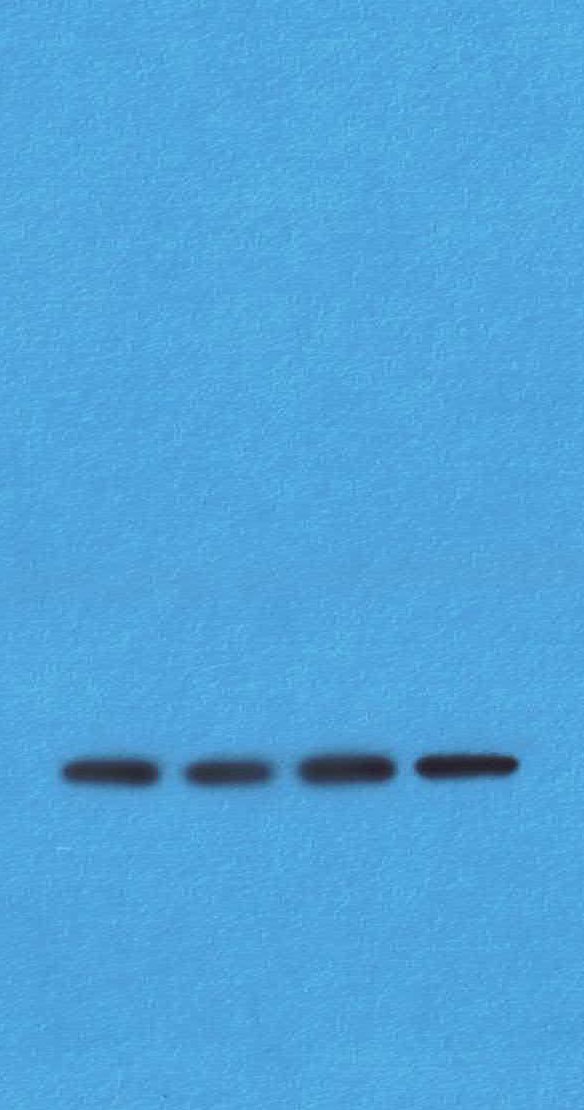

Supplement: Supplementary file 10 — Source data Fig. 2 [file 44318_2024_95_MOESM10_ESM.zip › SD Figure 2/2A/Crk4 GRA7.tif]

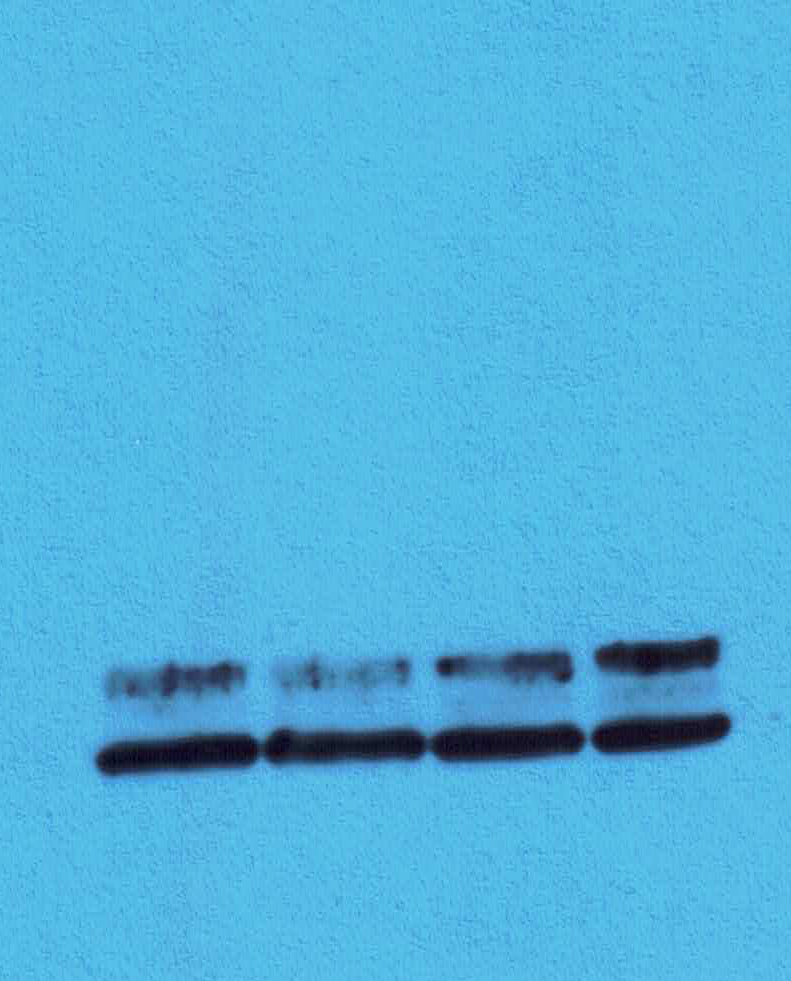

Supplement: Supplementary file 10 — Source data Fig. 2 [file 44318_2024_95_MOESM10_ESM.zip › SD Figure 2/2A/Crk6 GRA7.tif]

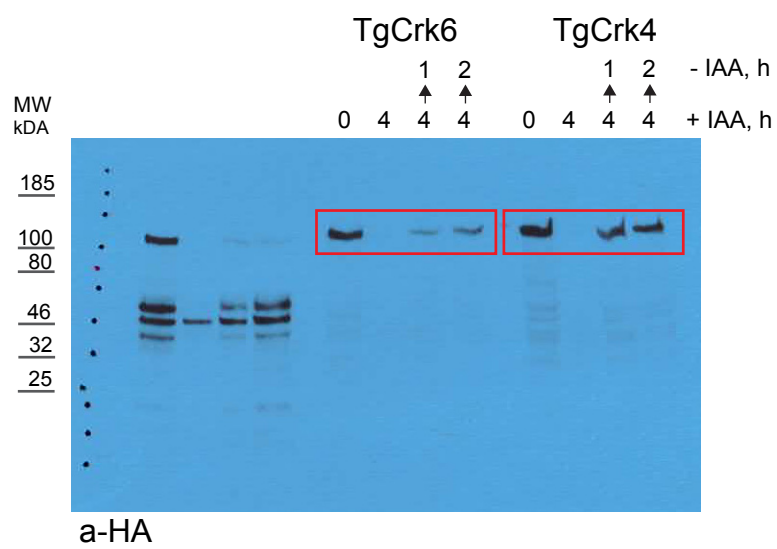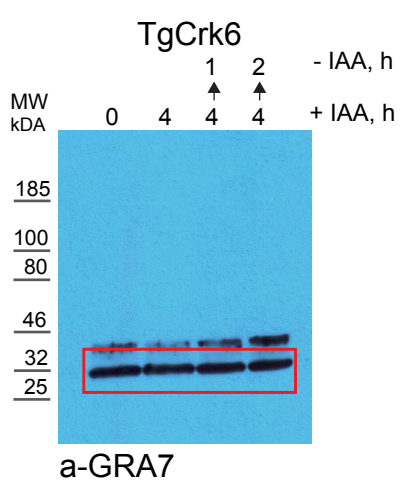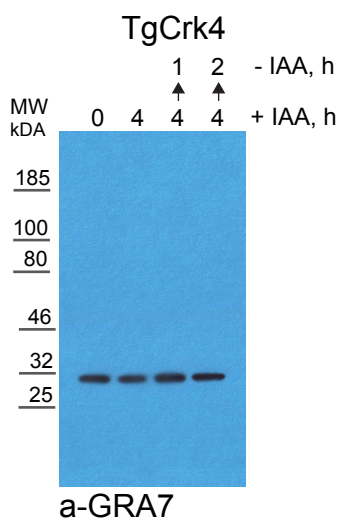

Supplement: Supplementary file 10 — Source data Fig. 2 [file 44318_2024_95_MOESM10_ESM.zip › SD Figure 2/2A/readme.pdf]

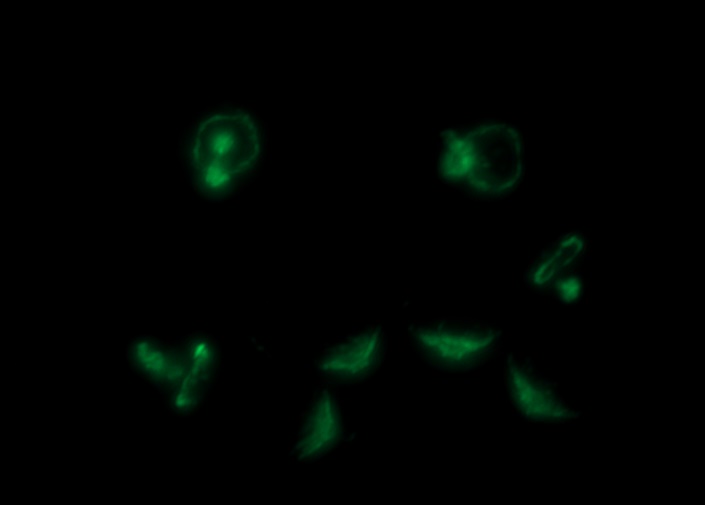

Supplement: Supplementary file 10 — Source data Fig. 2 [file 44318_2024_95_MOESM10_ESM.zip › SD Figure 2/2F/Crk4 +aux 4h MORN1.jpg]

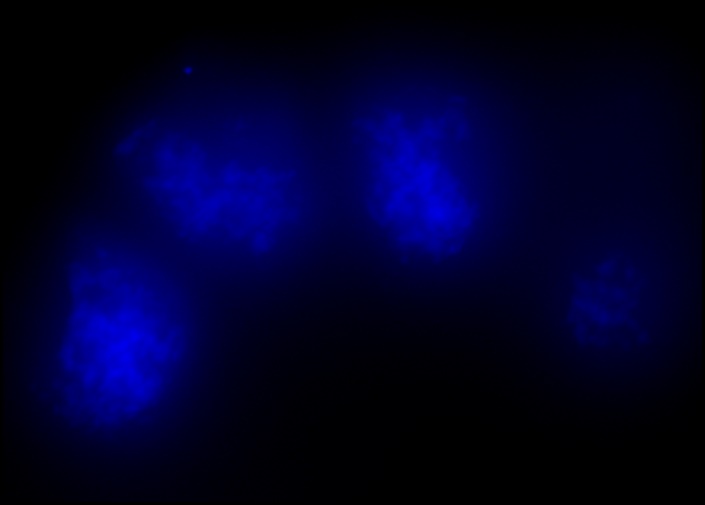

Supplement: Supplementary file 10 — Source data Fig. 2 [file 44318_2024_95_MOESM10_ESM.zip › SD Figure 2/2F/Crk4 +aux 4h DAPI.jpg]

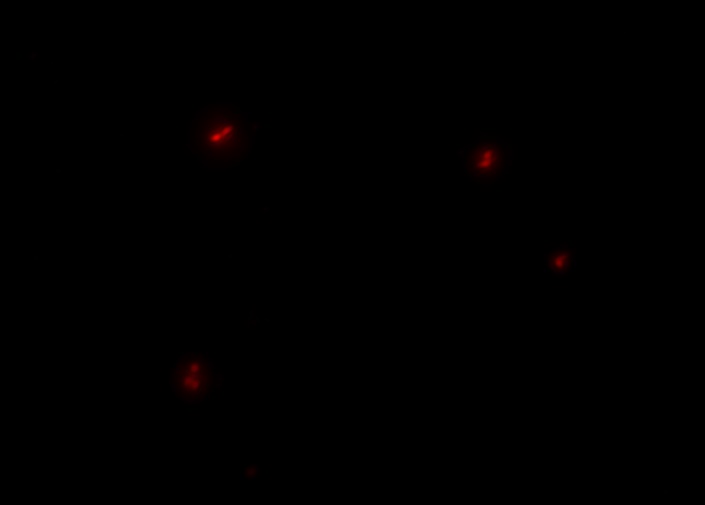

Supplement: Supplementary file 10 — Source data Fig. 2 [file 44318_2024_95_MOESM10_ESM.zip › SD Figure 2/2F/Crk4 +aux 4h centrin.jpg]

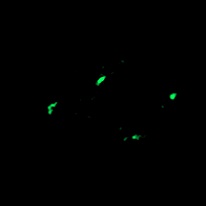

Supplement: Supplementary file 10 — Source data Fig. 2 [file 44318_2024_95_MOESM10_ESM.zip › SD Figure 2/2J/Crk4+CEP250L-1 +4h aux centrin.jpg]

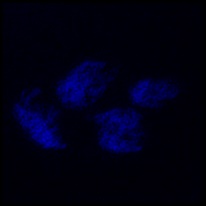

Supplement: Supplementary file 10 — Source data Fig. 2 [file 44318_2024_95_MOESM10_ESM.zip › SD Figure 2/2J/Crk4+CEP250L-1 +4h aux DAPI.jpg]

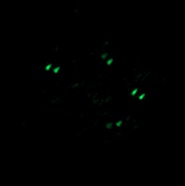

Supplement: Supplementary file 10 — Source data Fig. 2 [file 44318_2024_95_MOESM10_ESM.zip › SD Figure 2/2J/Crk4+CEP250L-1 - aux centrin panel-2.jpg]

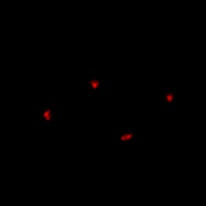

Supplement: Supplementary file 10 — Source data Fig. 2 [file 44318_2024_95_MOESM10_ESM.zip › SD Figure 2/2J/Crk4+CEP250L-1 +4h aux myc.jpg]

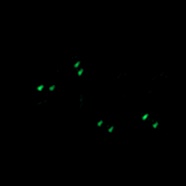

Supplement: Supplementary file 10 — Source data Fig. 2 [file 44318_2024_95_MOESM10_ESM.zip › SD Figure 2/2J/Crk4+CEP250L-1 - aux centrin panel-3.jpg]

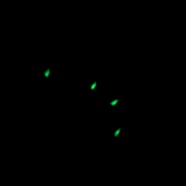

Supplement: Supplementary file 10 — Source data Fig. 2 [file 44318_2024_95_MOESM10_ESM.zip › SD Figure 2/2J/Crk4+CEP250L-1 - aux centrin panel-1.jpg]

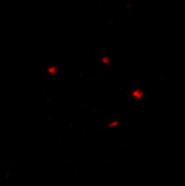

Supplement: Supplementary file 10 — Source data Fig. 2 [file 44318_2024_95_MOESM10_ESM.zip › SD Figure 2/2J/Crk4+CEP250L-1 - aux myc panel-2.jpg]

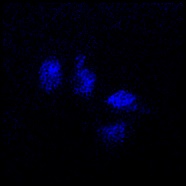

Supplement: Supplementary file 10 — Source data Fig. 2 [file 44318_2024_95_MOESM10_ESM.zip › SD Figure 2/2J/Crk4+CEP250L-1 - aux DAPI panel-1.jpg]

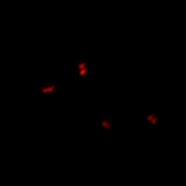

Supplement: Supplementary file 10 — Source data Fig. 2 [file 44318_2024_95_MOESM10_ESM.zip › SD Figure 2/2J/Crk4+CEP250L-1 - aux myc panel-3.jpg]

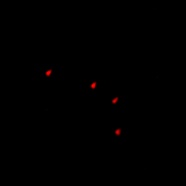

Supplement: Supplementary file 10 — Source data Fig. 2 [file 44318_2024_95_MOESM10_ESM.zip › SD Figure 2/2J/Crk4+CEP250L-1 - aux myc panel-1.jpg]

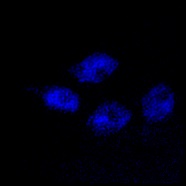

Supplement: Supplementary file 10 — Source data Fig. 2 [file 44318_2024_95_MOESM10_ESM.zip › SD Figure 2/2J/Crk4+CEP250L-1 - aux DAPI panel-3.jpg]

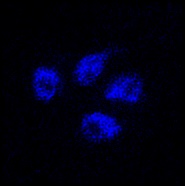

Supplement: Supplementary file 10 — Source data Fig. 2 [file 44318_2024_95_MOESM10_ESM.zip › SD Figure 2/2J/Crk4+CEP250L-1 - aux DAPI panel-2.jpg]

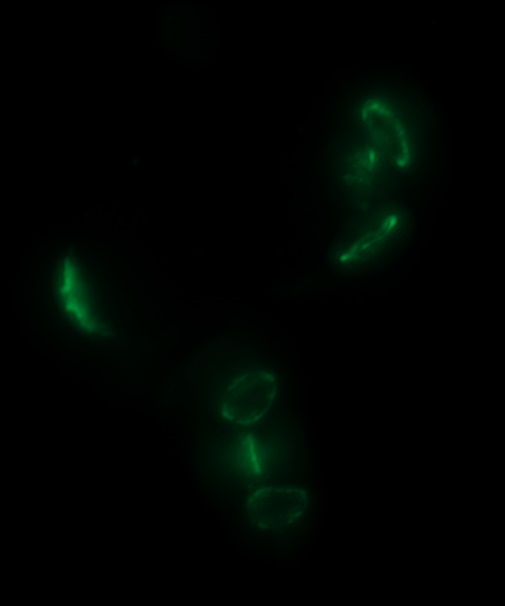

Supplement: Supplementary file 10 — Source data Fig. 2 [file 44318_2024_95_MOESM10_ESM.zip › SD Figure 2/2E/Crk4 -aux 4h MORN1.jpg]

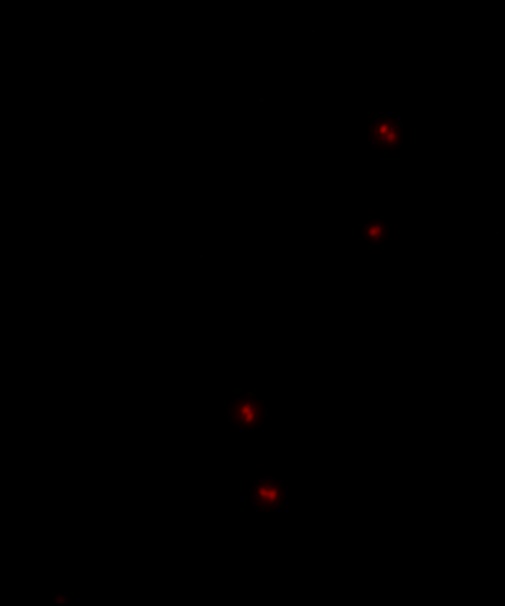

Supplement: Supplementary file 10 — Source data Fig. 2 [file 44318_2024_95_MOESM10_ESM.zip › SD Figure 2/2E/Crk4 -aux 4h centrin.jpg]

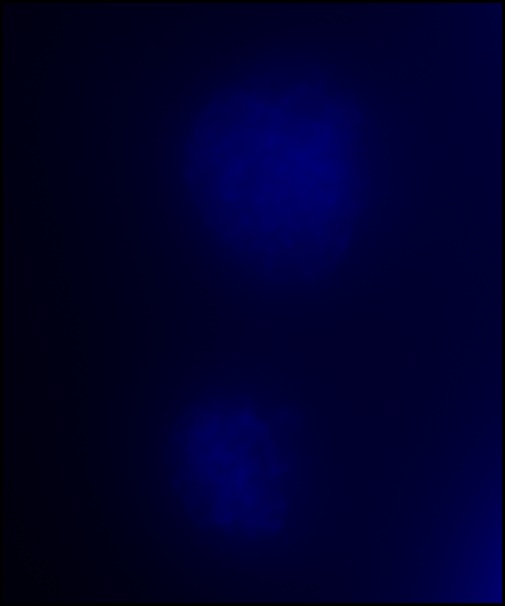

Supplement: Supplementary file 10 — Source data Fig. 2 [file 44318_2024_95_MOESM10_ESM.zip › SD Figure 2/2E/Crk4 -aux 4h DAPI.jpg]

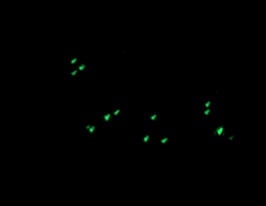

Supplement: Supplementary file 11 — Source data Fig. 3 [file 44318_2024_95_MOESM11_ESM.zip › SD Figure 3/3B/Crk4 1h post-recovery centrin panel-2.jpg]

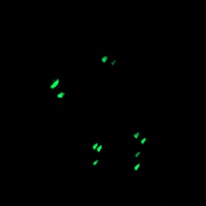

Supplement: Supplementary file 11 — Source data Fig. 3 [file 44318_2024_95_MOESM11_ESM.zip › SD Figure 3/3B/Crk4 1h post-recovery centrin panel-1.jpg]

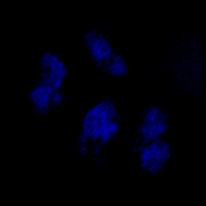

Supplement: Supplementary file 11 — Source data Fig. 3 [file 44318_2024_95_MOESM11_ESM.zip › SD Figure 3/3B/Crk4 1h post-recovery DAPI panel-1.jpg]

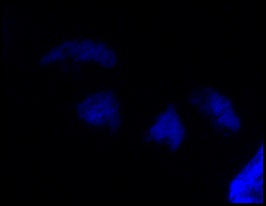

Supplement: Supplementary file 11 — Source data Fig. 3 [file 44318_2024_95_MOESM11_ESM.zip › SD Figure 3/3B/Crk4 1h post-recovery DAPI panel-2.jpg]

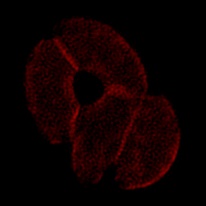

Supplement: Supplementary file 11 — Source data Fig. 3 [file 44318_2024_95_MOESM11_ESM.zip › SD Figure 3/3B/Crk4 1h post-recovery IMC1 panel-1.jpg]

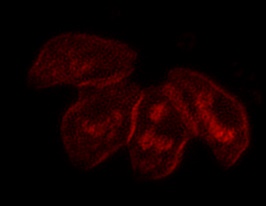

Supplement: Supplementary file 11 — Source data Fig. 3 [file 44318_2024_95_MOESM11_ESM.zip › SD Figure 3/3B/Crk4 1h post-recovery IMC1 panel-2.jpg]

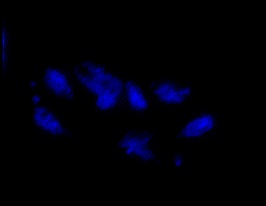

Supplement: Supplementary file 11 — Source data Fig. 3 [file 44318_2024_95_MOESM11_ESM.zip › SD Figure 3/3C/Crk4 2h post-recovery DAPI panel-1.jpg]

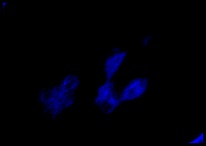

Supplement: Supplementary file 11 — Source data Fig. 3 [file 44318_2024_95_MOESM11_ESM.zip › SD Figure 3/3C/Crk4 2h post-recovery DAPI panel-2.jpg]

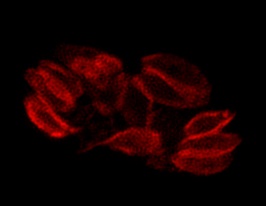

Supplement: Supplementary file 11 — Source data Fig. 3 [file 44318_2024_95_MOESM11_ESM.zip › SD Figure 3/3C/Crk4 2h post-recovery IMC1 panel-1.jpg]

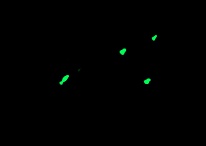

Supplement: Supplementary file 11 — Source data Fig. 3 [file 44318_2024_95_MOESM11_ESM.zip › SD Figure 3/3C/Crk4 2h post-recovery centrin panel-2.jpg]

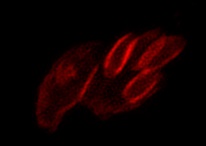

Supplement: Supplementary file 11 — Source data Fig. 3 [file 44318_2024_95_MOESM11_ESM.zip › SD Figure 3/3C/Crk4 2h post-recovery IMC1 panel-2.jpg]

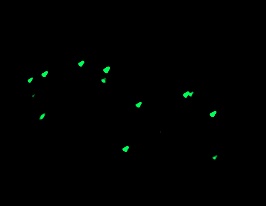

Supplement: Supplementary file 11 — Source data Fig. 3 [file 44318_2024_95_MOESM11_ESM.zip › SD Figure 3/3C/Crk4 2h post-recovery centrin panel-1.jpg]

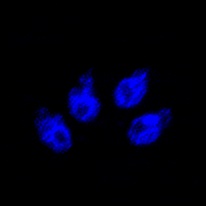

Supplement: Supplementary file 11 — Source data Fig. 3 [file 44318_2024_95_MOESM11_ESM.zip › SD Figure 3/3A/Crk4 4h block DAPI panel-1.jpg]

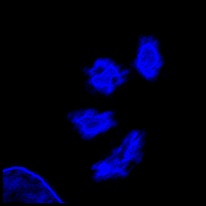

Supplement: Supplementary file 11 — Source data Fig. 3 [file 44318_2024_95_MOESM11_ESM.zip › SD Figure 3/3A/Crk4 4h block DAPI panel-2.jpg]

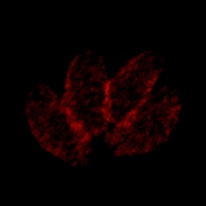

Supplement: Supplementary file 11 — Source data Fig. 3 [file 44318_2024_95_MOESM11_ESM.zip › SD Figure 3/3A/Crk4 4h block IMC1 panel-1.jpg]

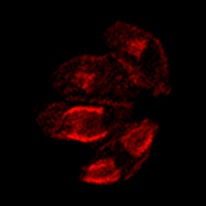

Supplement: Supplementary file 11 — Source data Fig. 3 [file 44318_2024_95_MOESM11_ESM.zip › SD Figure 3/3A/Crk4 4h block IMC1 panel-2.jpg]

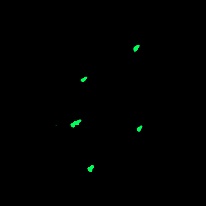

Supplement: Supplementary file 11 — Source data Fig. 3 [file 44318_2024_95_MOESM11_ESM.zip › SD Figure 3/3A/Crk4 4h block centrin panel-2.jpg]

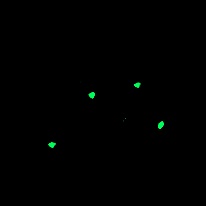

Supplement: Supplementary file 11 — Source data Fig. 3 [file 44318_2024_95_MOESM11_ESM.zip › SD Figure 3/3A/Crk4 4h block centrin panel-1.jpg]

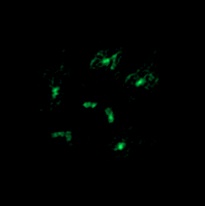

Supplement: Supplementary file 13 — Source data Fig. 6 [file 44318_2024_95_MOESM13_ESM.zip › SD Figure 6/6F/iRD1 MORN1 mitosis.jpg]

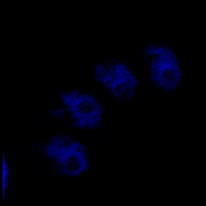

Supplement: Supplementary file 13 — Source data Fig. 6 [file 44318_2024_95_MOESM13_ESM.zip › SD Figure 6/6F/iRD1 DAPI S-phase.jpg]

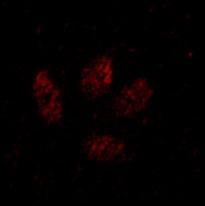

Supplement: Supplementary file 13 — Source data Fig. 6 [file 44318_2024_95_MOESM13_ESM.zip › SD Figure 6/6F/iRD1 HA mitosis.jpg]

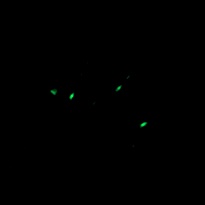

Supplement: Supplementary file 13 — Source data Fig. 6 [file 44318_2024_95_MOESM13_ESM.zip › SD Figure 6/6F/iRD1 centrin G1.jpg]

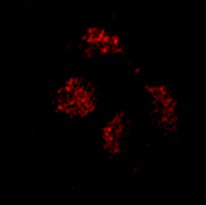

Supplement: Supplementary file 13 — Source data Fig. 6 [file 44318_2024_95_MOESM13_ESM.zip › SD Figure 6/6F/iRD1 HA budding panel-1.jpg]

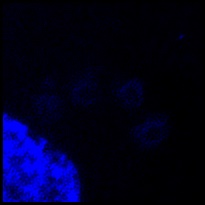

Supplement: Supplementary file 13 — Source data Fig. 6 [file 44318_2024_95_MOESM13_ESM.zip › SD Figure 6/6F/iRD1 DAPI G1 HA.jpg]

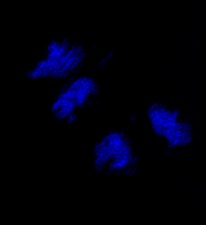

Supplement: Supplementary file 13 — Source data Fig. 6 [file 44318_2024_95_MOESM13_ESM.zip › SD Figure 6/6F/iRD1 DAPI budding panel-2.jpg]

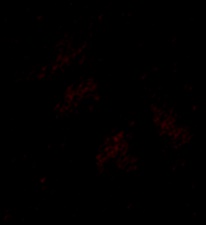

Supplement: Supplementary file 13 — Source data Fig. 6 [file 44318_2024_95_MOESM13_ESM.zip › SD Figure 6/6F/iRD1 HA budding panel-2.jpg]

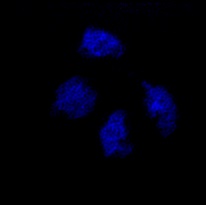

Supplement: Supplementary file 13 — Source data Fig. 6 [file 44318_2024_95_MOESM13_ESM.zip › SD Figure 6/6F/iRD1 DAPI budding panel-1.jpg]

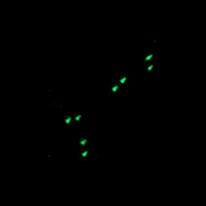

Supplement: Supplementary file 13 — Source data Fig. 6 [file 44318_2024_95_MOESM13_ESM.zip › SD Figure 6/6F/iRD1 centrin S-phase.jpg]

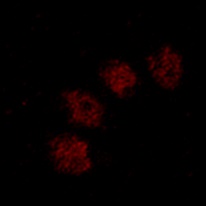

Supplement: Supplementary file 13 — Source data Fig. 6 [file 44318_2024_95_MOESM13_ESM.zip › SD Figure 6/6F/iRD1 HA S-phase.jpg]

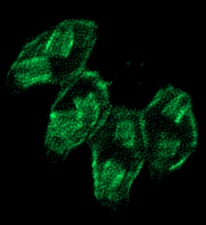

Supplement: Supplementary file 13 — Source data Fig. 6 [file 44318_2024_95_MOESM13_ESM.zip › SD Figure 6/6F/iRD1 IMC1 budding panel-2.jpg]

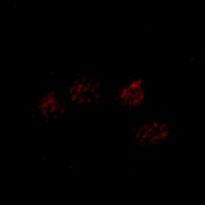

Supplement: Supplementary file 13 — Source data Fig. 6 [file 44318_2024_95_MOESM13_ESM.zip › SD Figure 6/6F/iRD1 HA G1.jpg]

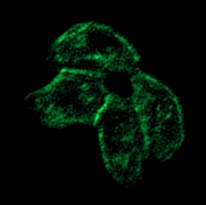

Supplement: Supplementary file 13 — Source data Fig. 6 [file 44318_2024_95_MOESM13_ESM.zip › SD Figure 6/6F/iRD1 IMC1 budding panel-1.jpg]

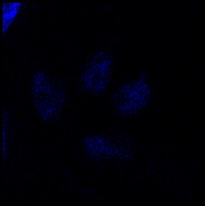

Supplement: Supplementary file 13 — Source data Fig. 6 [file 44318_2024_95_MOESM13_ESM.zip › SD Figure 6/6F/iRD1 DAPI mitosis.jpg]

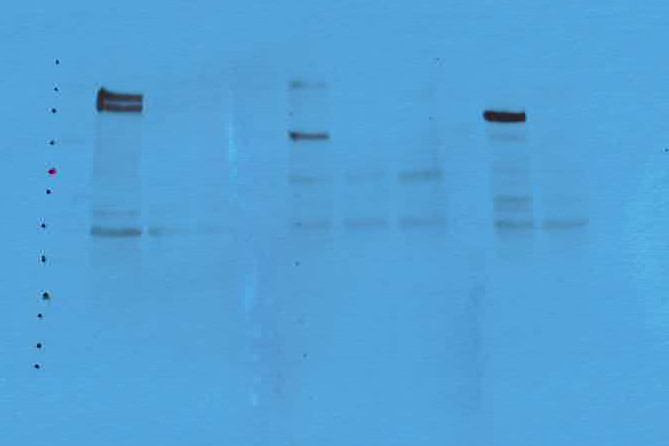

Supplement: Supplementary file 13 — Source data Fig. 6 [file 44318_2024_95_MOESM13_ESM.zip › SD Figure 6/6E/iRD1 HA.tif]

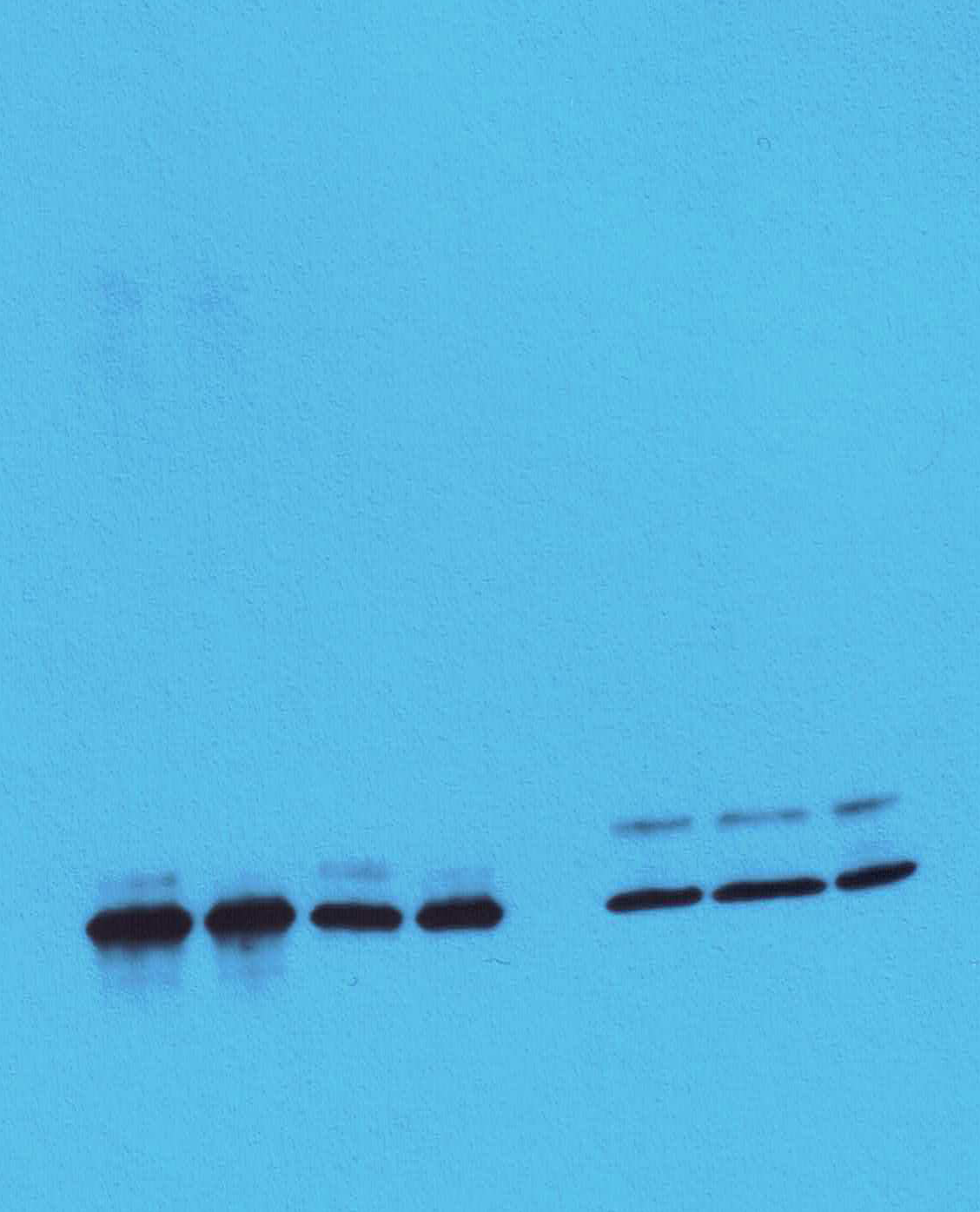

Supplement: Supplementary file 13 — Source data Fig. 6 [file 44318_2024_95_MOESM13_ESM.zip › SD Figure 6/6E/iRD1 GRA7.tif]
