# Supplementary figures and images for: The Crk4-Cyc4 complex regulates G2/M transition in Toxoplasma gondii (part 2 of 2)
Source: EMBO J. 2024 Apr 10;43(11):2094–126. doi: 10.1038/s44318-024-00095-4 (PMC11148040; doi:10.1038/s44318-024-00095-4)

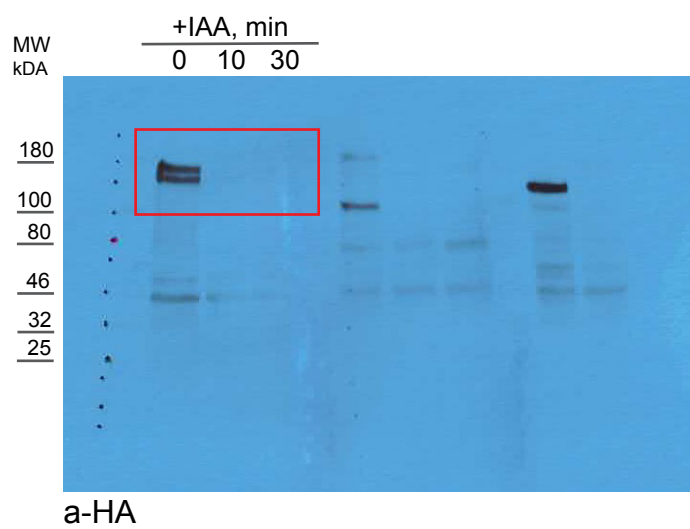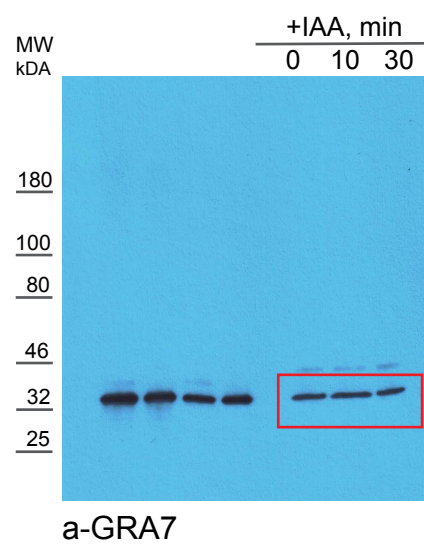

Supplement: Supplementary file 13 — Source data Fig. 6 [file 44318_2024_95_MOESM13_ESM.zip › SD Figure 6/6E/readme.pdf]

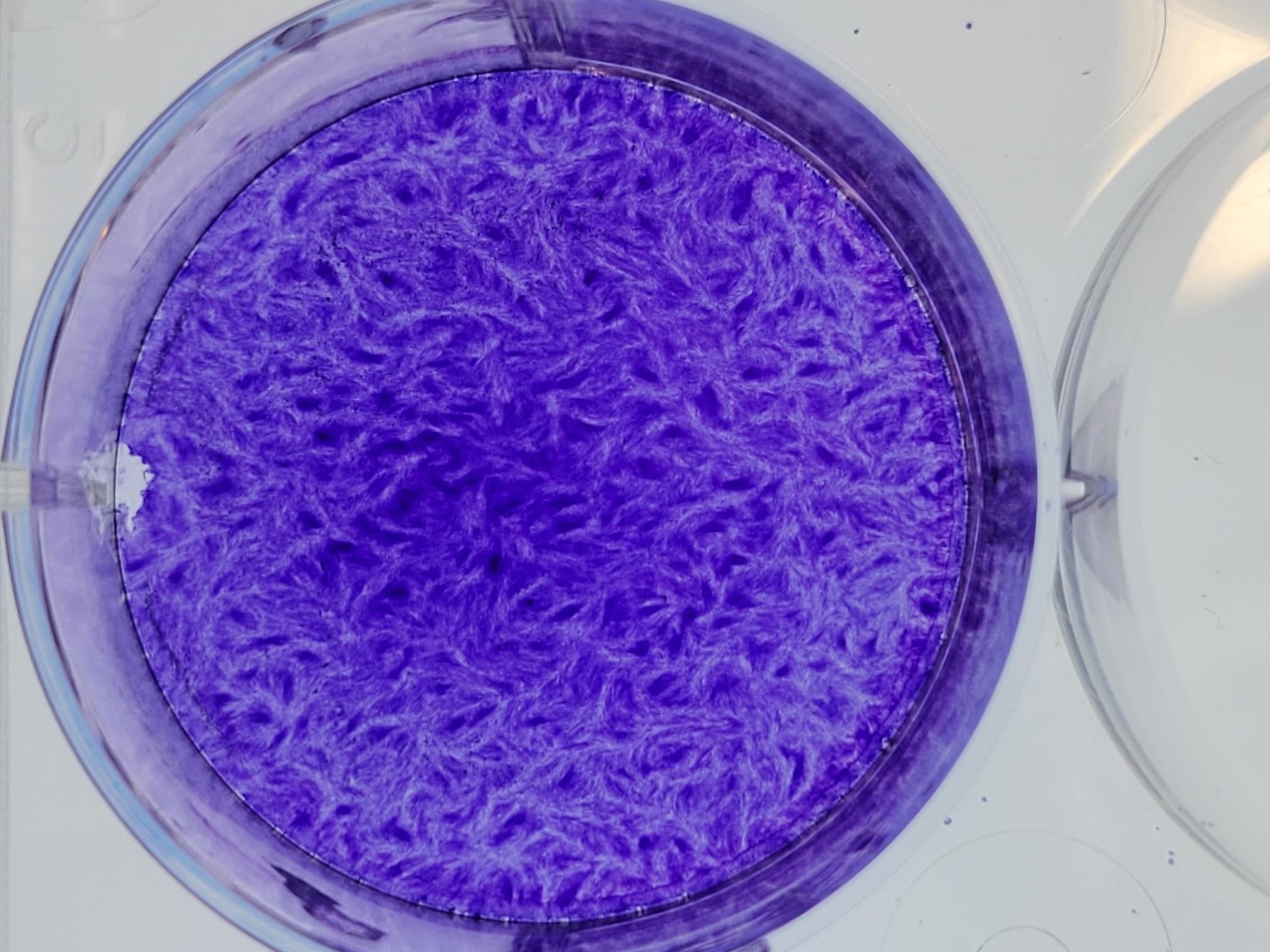

Supplement: Supplementary file 13 — Source data Fig. 6 [file 44318_2024_95_MOESM13_ESM.zip › SD Figure 6/6D/iRD1 PA +IAA.jpg]

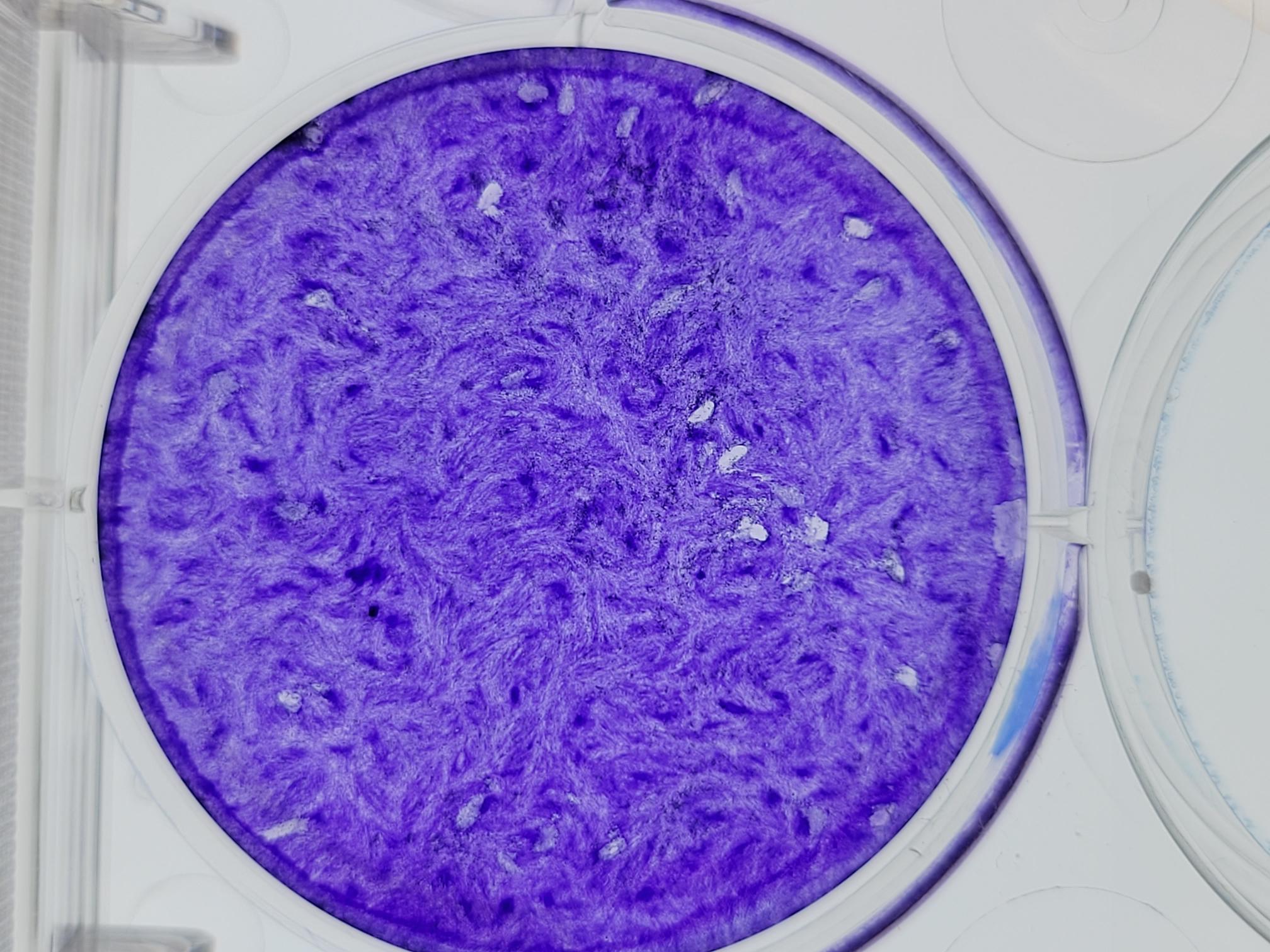

Supplement: Supplementary file 13 — Source data Fig. 6 [file 44318_2024_95_MOESM13_ESM.zip › SD Figure 6/6D/iRD1 PA -IAA.jpg]

RH TgiRD1<sup>AID-HA</sup>

-IAA

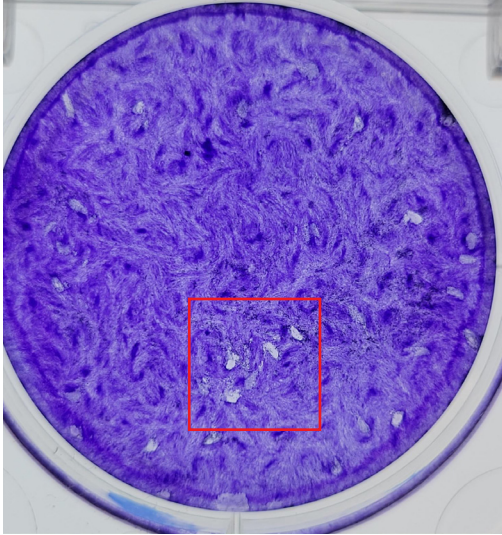

+IAA

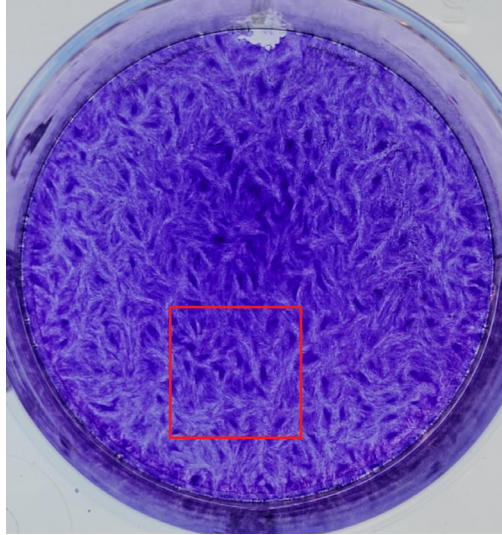

Supplement: Supplementary file 13 — Source data Fig. 6 [file 44318_2024_95_MOESM13_ESM.zip › SD Figure 6/6D/readme.pdf]

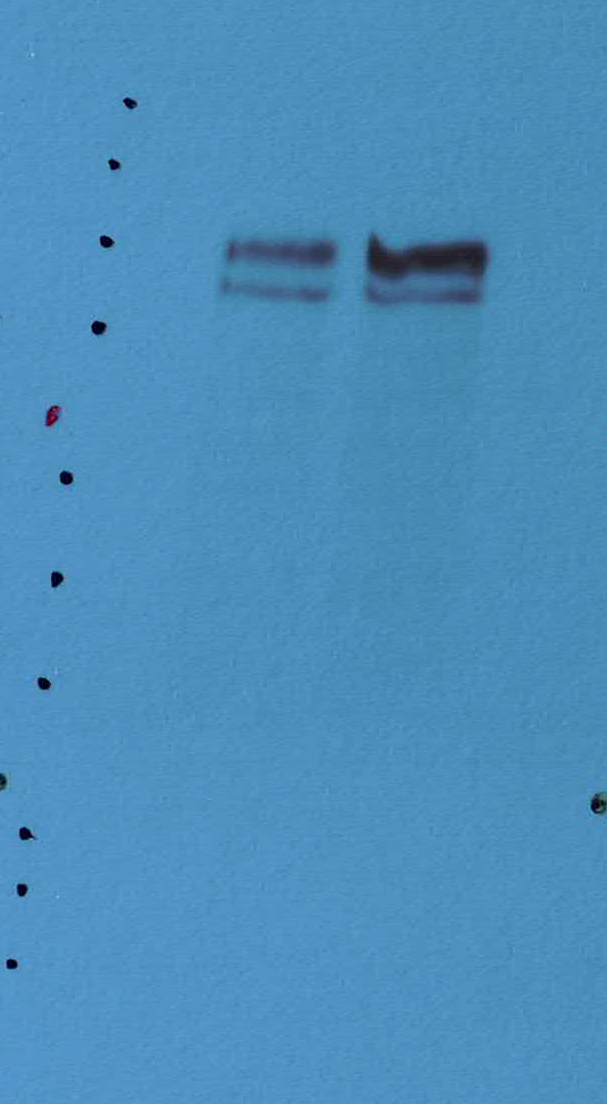

Supplement: Supplementary file 14 — Source data Fig. 7 [file 44318_2024_95_MOESM14_ESM.zip › SD Figure 7/7J/iRD1 myc.tif]

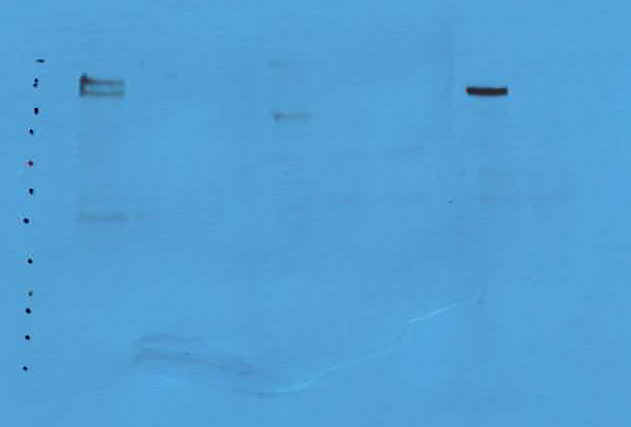

Supplement: Supplementary file 14 — Source data Fig. 7 [file 44318_2024_95_MOESM14_ESM.zip › SD Figure 7/7J/iRD1 HA.tif]

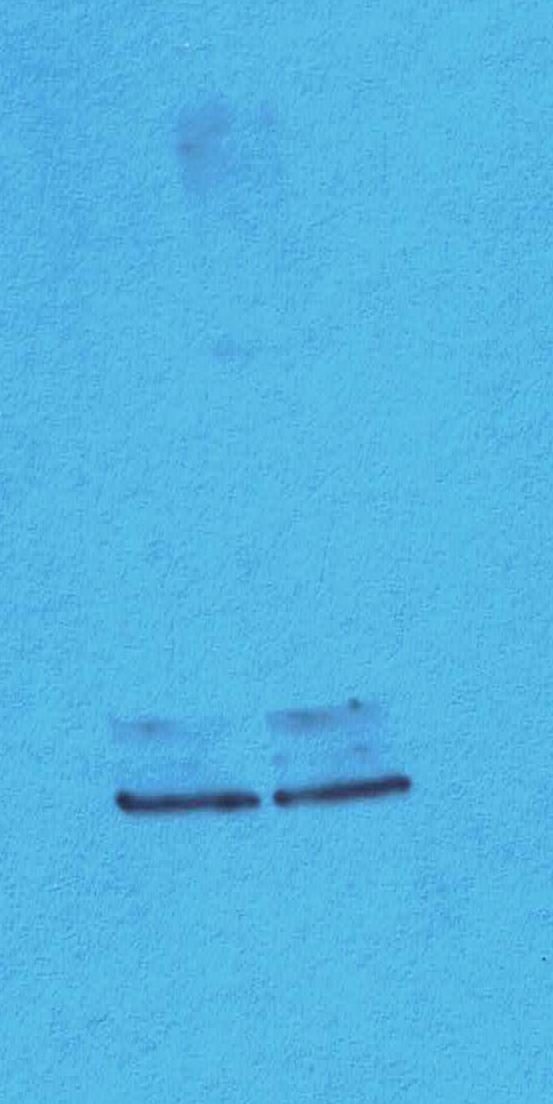

Supplement: Supplementary file 14 — Source data Fig. 7 [file 44318_2024_95_MOESM14_ESM.zip › SD Figure 7/7J/iRD1 GRA7.tif]

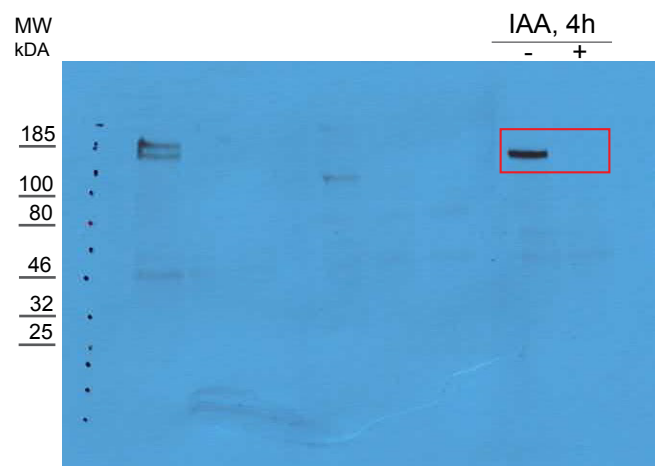

a-HA

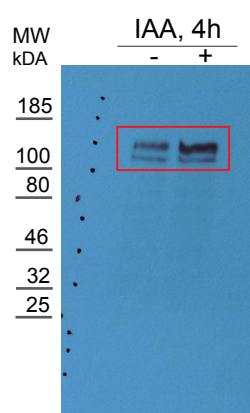

a-myc

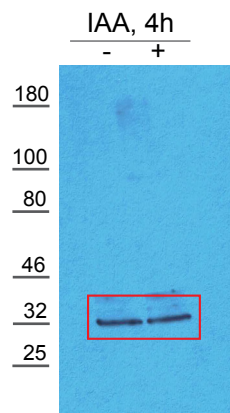

a-GRA7

Supplement: Supplementary file 14 — Source data Fig. 7 [file 44318_2024_95_MOESM14_ESM.zip › SD Figure 7/7J/readme.pdf]

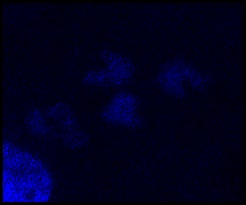

Supplement: Supplementary file 14 — Source data Fig. 7 [file 44318_2024_95_MOESM14_ESM.zip › SD Figure 7/7C/iRD1 -aux 4h DAPI.jpg]

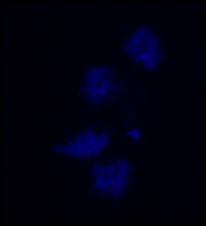

Supplement: Supplementary file 14 — Source data Fig. 7 [file 44318_2024_95_MOESM14_ESM.zip › SD Figure 7/7C/iRD1 +aux 4h DAPI.jpg]

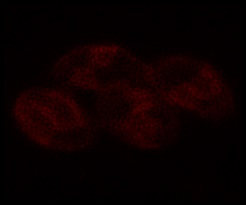

Supplement: Supplementary file 14 — Source data Fig. 7 [file 44318_2024_95_MOESM14_ESM.zip › SD Figure 7/7C/iRD1 -aux 4h IMC1.jpg]

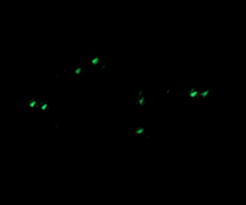

Supplement: Supplementary file 14 — Source data Fig. 7 [file 44318_2024_95_MOESM14_ESM.zip › SD Figure 7/7C/iRD1 -aux 4h centrin.jpg]

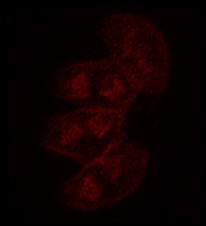

Supplement: Supplementary file 14 — Source data Fig. 7 [file 44318_2024_95_MOESM14_ESM.zip › SD Figure 7/7C/iRD1 +aux 4h IMC1.jpg]

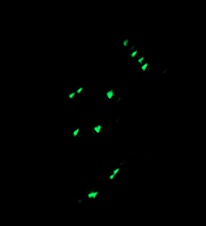

Supplement: Supplementary file 14 — Source data Fig. 7 [file 44318_2024_95_MOESM14_ESM.zip › SD Figure 7/7C/iRD1 +aux 4h centrin.jpg]

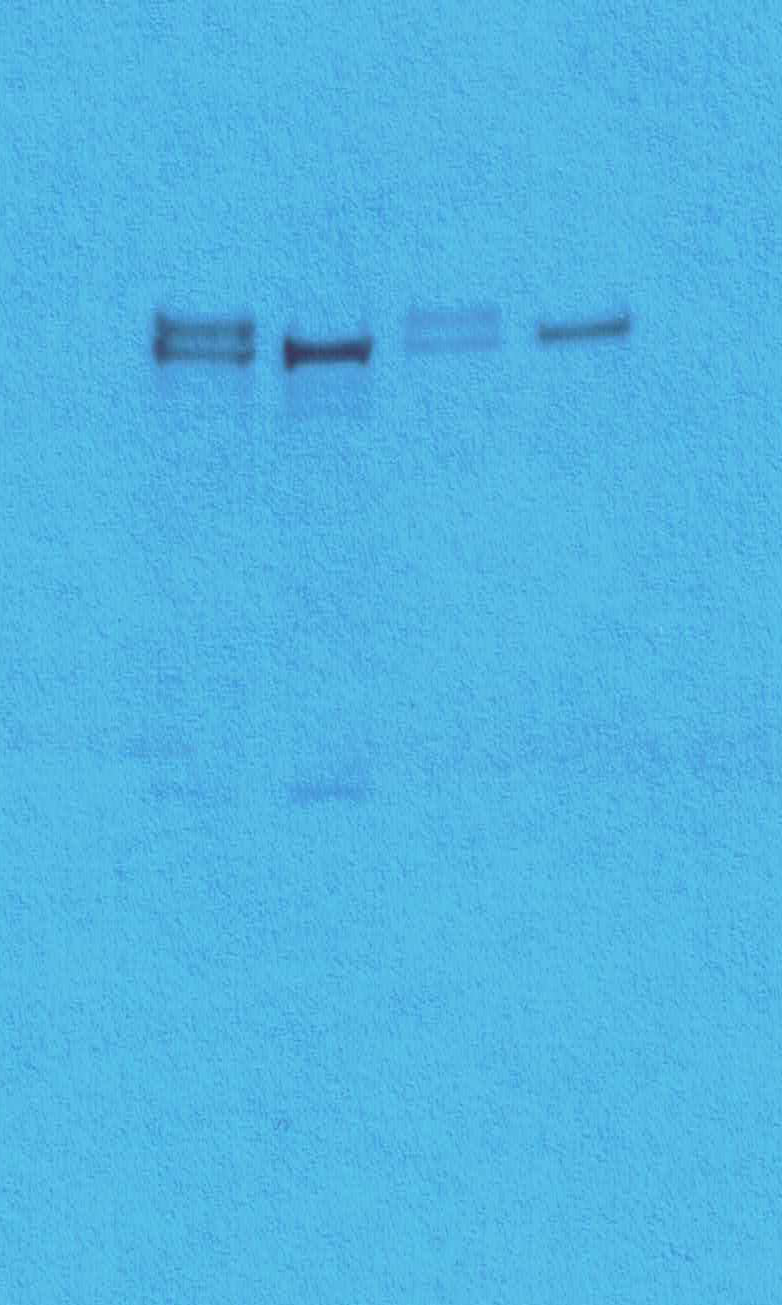

Supplement: Supplementary file 14 — Source data Fig. 7 [file 44318_2024_95_MOESM14_ESM.zip › SD Figure 7/7I/iRD1 myc.tif]

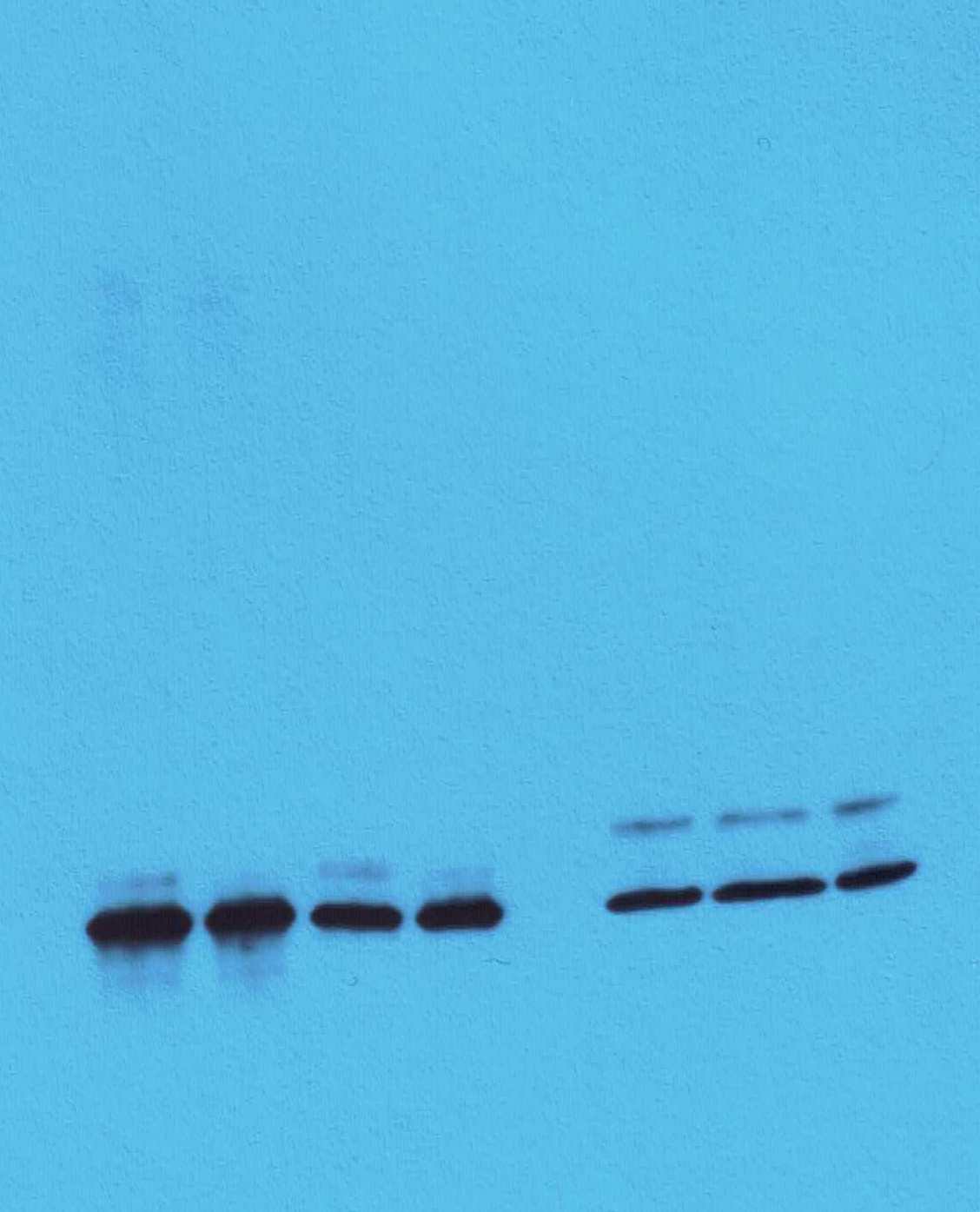

Supplement: Supplementary file 14 — Source data Fig. 7 [file 44318_2024_95_MOESM14_ESM.zip › SD Figure 7/7I/iRD1 GRA7.tif]

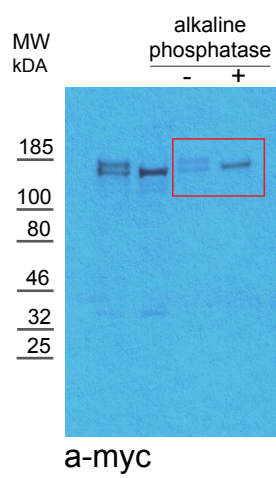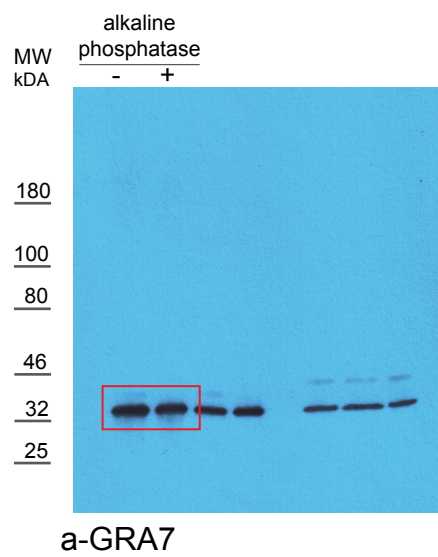

Supplement: Supplementary file 14 — Source data Fig. 7 [file 44318_2024_95_MOESM14_ESM.zip › SD Figure 7/7I/readme.pdf]

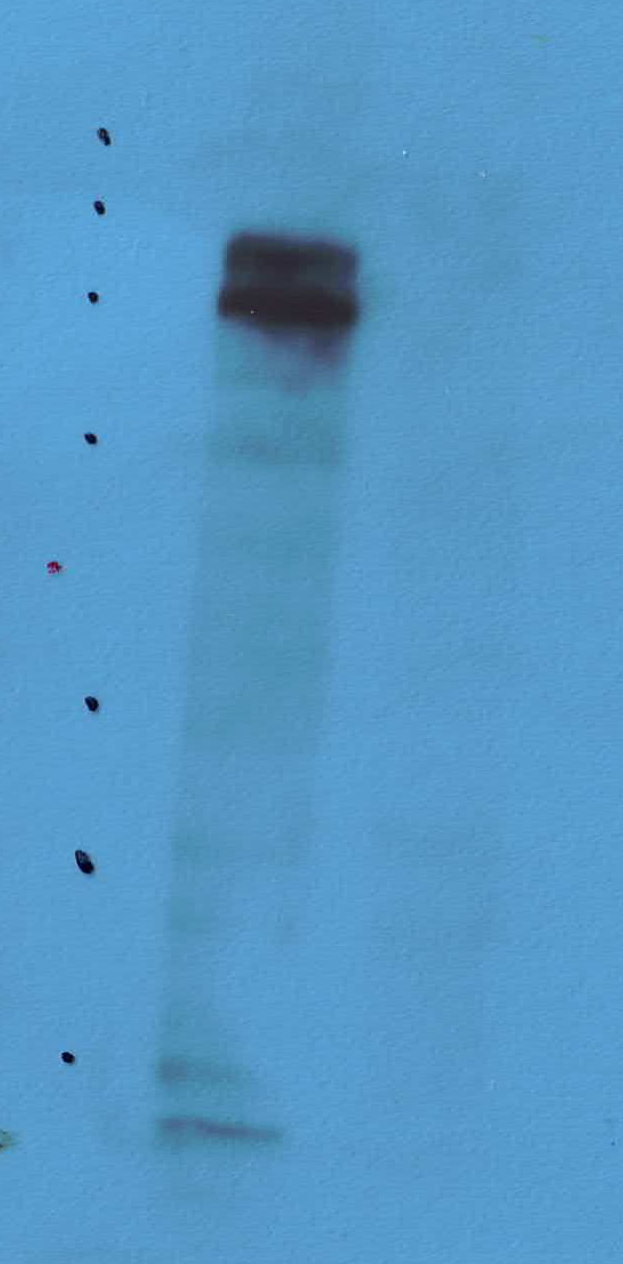

Supplement: Supplementary file 14 — Source data Fig. 7 [file 44318_2024_95_MOESM14_ESM.zip › SD Figure 7/7G/iRD1 HA.tif]

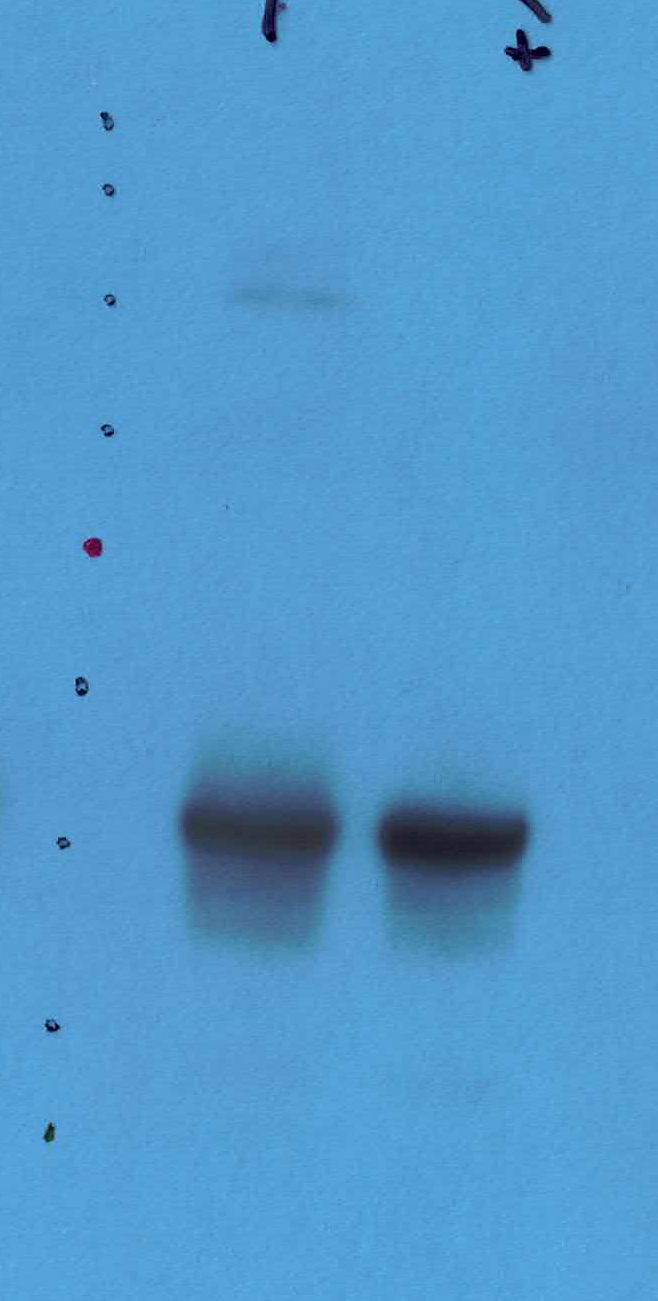

Supplement: Supplementary file 14 — Source data Fig. 7 [file 44318_2024_95_MOESM14_ESM.zip › SD Figure 7/7G/Tubulin.tif]

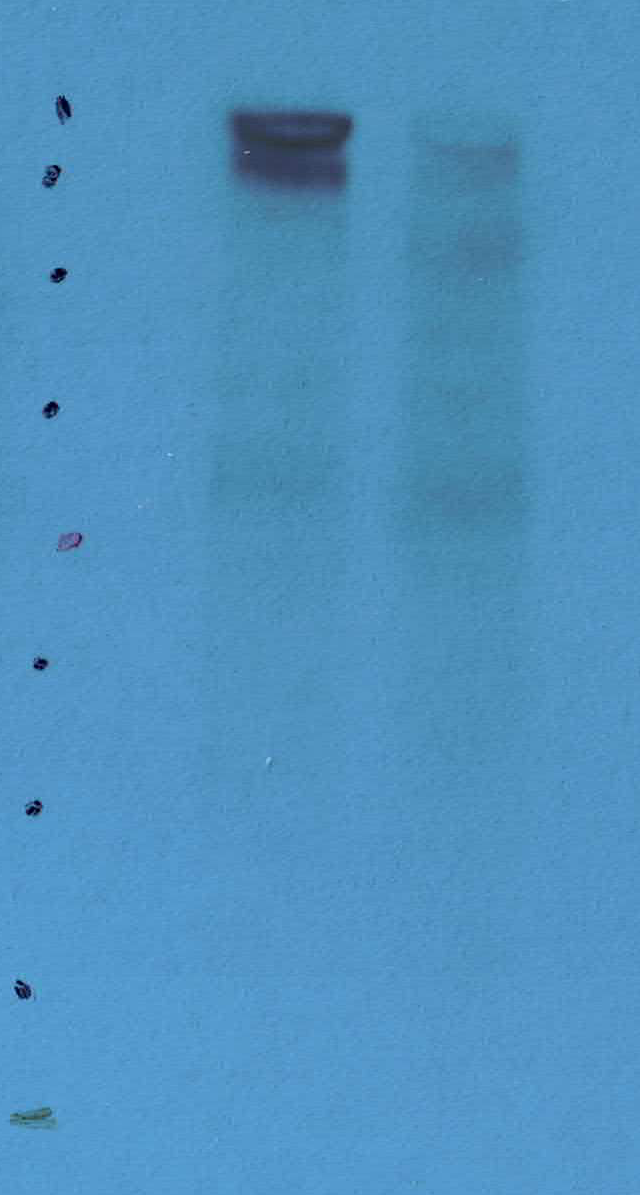

Supplement: Supplementary file 14 — Source data Fig. 7 [file 44318_2024_95_MOESM14_ESM.zip › SD Figure 7/7G/CEP250-L1 myc.tif]

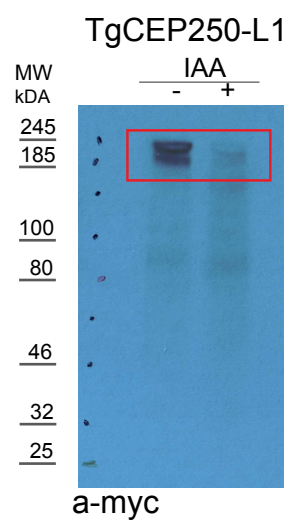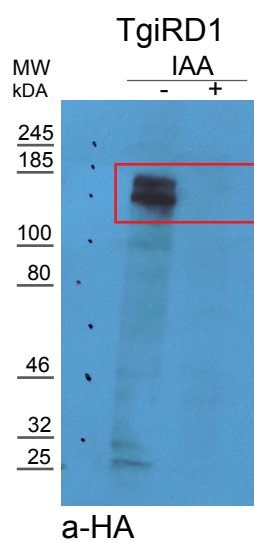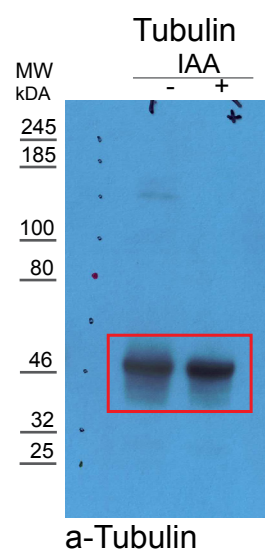

Supplement: Supplementary file 14 — Source data Fig. 7 [file 44318_2024_95_MOESM14_ESM.zip › SD Figure 7/7G/readme.pdf]

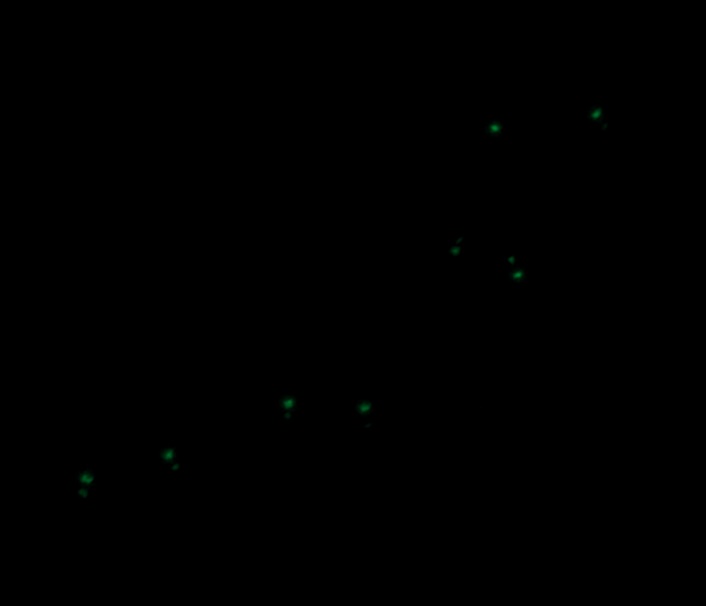

Supplement: Supplementary file 14 — Source data Fig. 7 [file 44318_2024_95_MOESM14_ESM.zip › SD Figure 7/7F/iRD1+CEP250L-1 no aux myc panel-2.jpg]

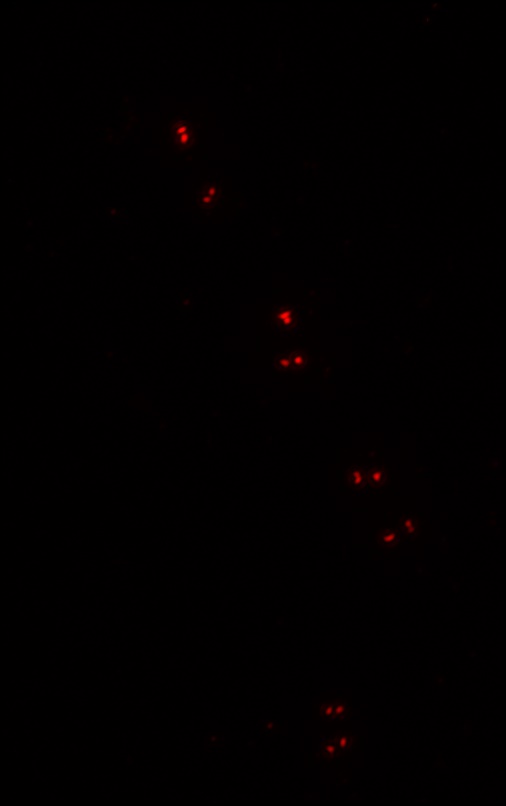

Supplement: Supplementary file 14 — Source data Fig. 7 [file 44318_2024_95_MOESM14_ESM.zip › SD Figure 7/7F/iRD1+CEP250L-1 4h aux centrin panel-1.jpg]

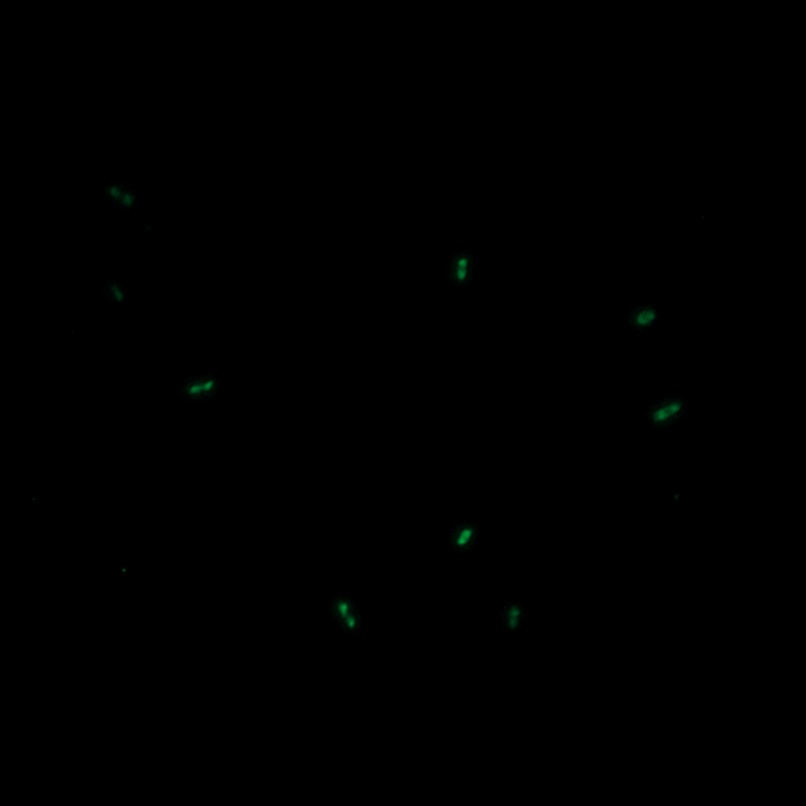

Supplement: Supplementary file 14 — Source data Fig. 7 [file 44318_2024_95_MOESM14_ESM.zip › SD Figure 7/7F/iRD1+CEP250L-1 no aux myc panel-1.jpg]

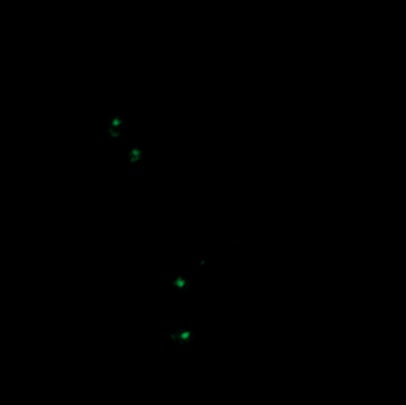

Supplement: Supplementary file 14 — Source data Fig. 7 [file 44318_2024_95_MOESM14_ESM.zip › SD Figure 7/7F/iRD1+CEP250L-1 4h aux myc panel-2.jpg]

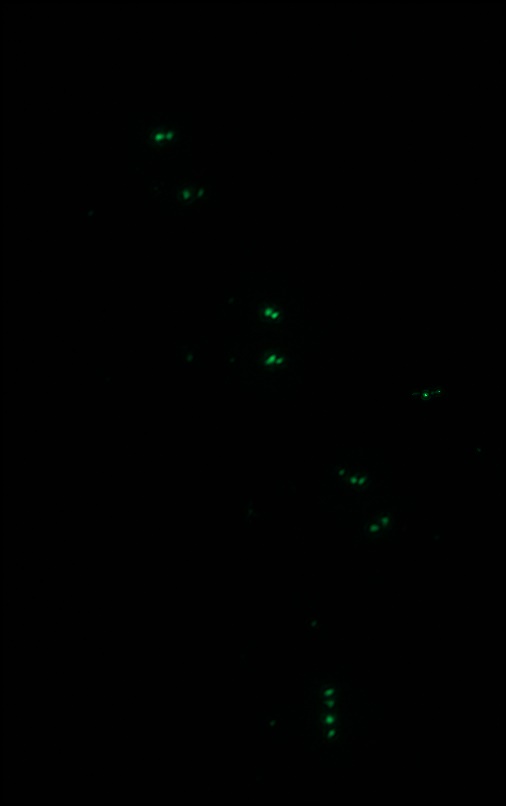

Supplement: Supplementary file 14 — Source data Fig. 7 [file 44318_2024_95_MOESM14_ESM.zip › SD Figure 7/7F/iRD1+CEP250L-1 4h aux myc panel-1.jpg]

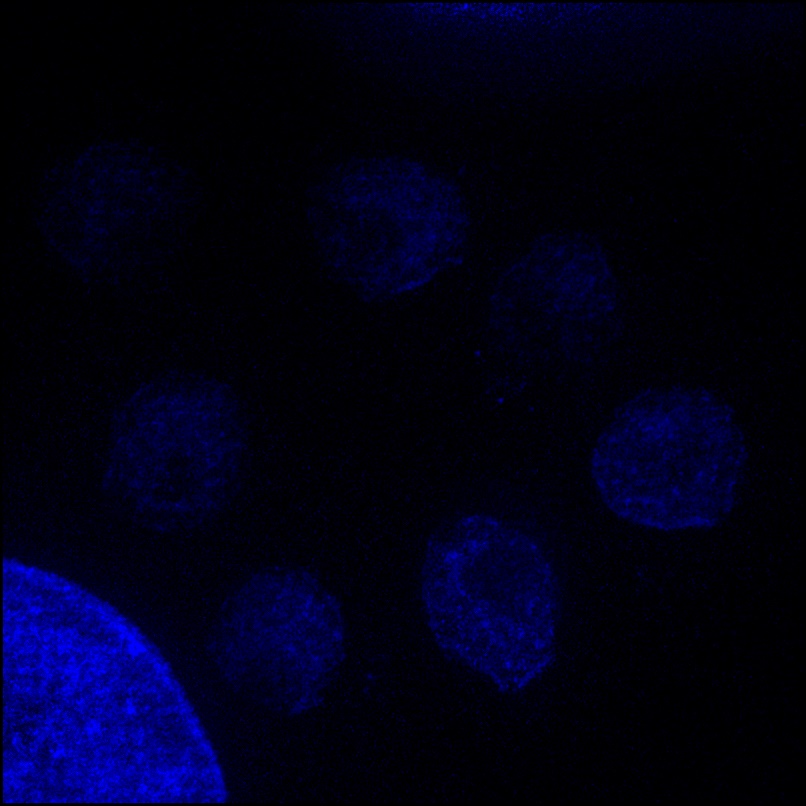

Supplement: Supplementary file 14 — Source data Fig. 7 [file 44318_2024_95_MOESM14_ESM.zip › SD Figure 7/7F/iRD1+CEP250L-1 no aux DAPI panel-1.jpg]

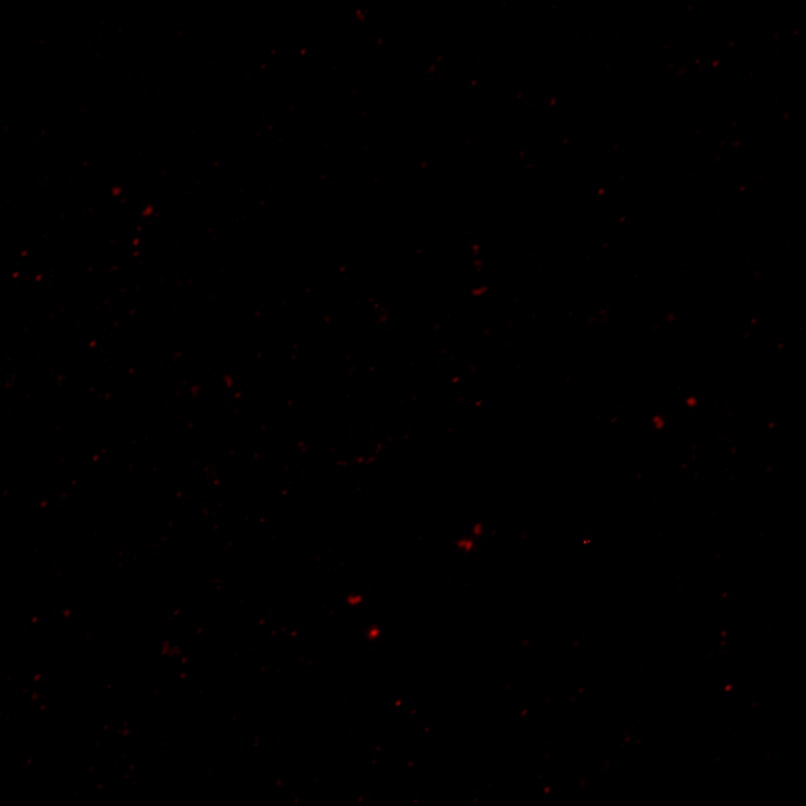

Supplement: Supplementary file 14 — Source data Fig. 7 [file 44318_2024_95_MOESM14_ESM.zip › SD Figure 7/7F/iRD1+CEP250L-1 no aux centrin panel-1.jpg]

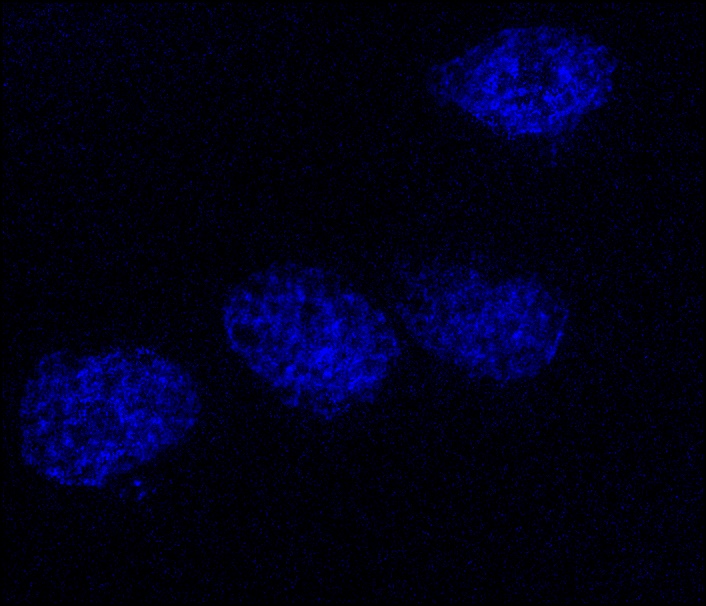

Supplement: Supplementary file 14 — Source data Fig. 7 [file 44318_2024_95_MOESM14_ESM.zip › SD Figure 7/7F/iRD1+CEP250L-1 no aux DAPI panel-2.jpg]

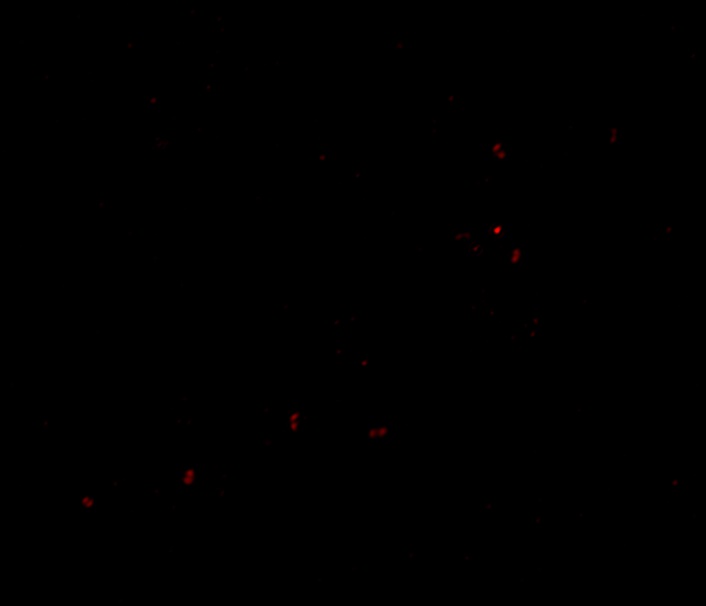

Supplement: Supplementary file 14 — Source data Fig. 7 [file 44318_2024_95_MOESM14_ESM.zip › SD Figure 7/7F/iRD1+CEP250L-1 no aux centrin panel-2.jpg]

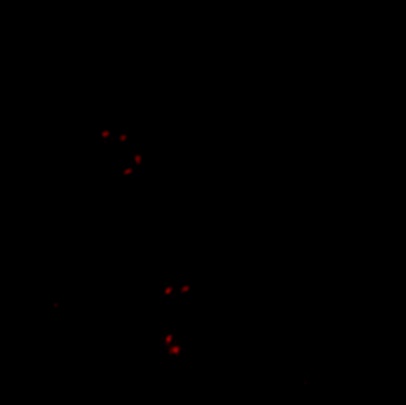

Supplement: Supplementary file 14 — Source data Fig. 7 [file 44318_2024_95_MOESM14_ESM.zip › SD Figure 7/7F/iRD1+CEP250L-1 4h aux centrin1 panel-2.jpg]

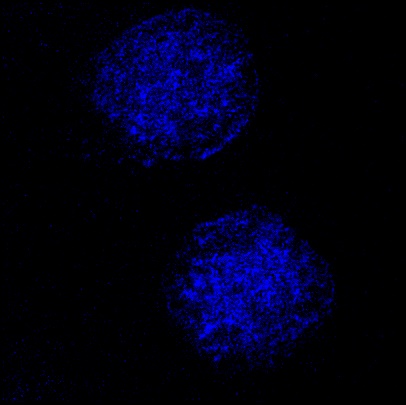

Supplement: Supplementary file 14 — Source data Fig. 7 [file 44318_2024_95_MOESM14_ESM.zip › SD Figure 7/7F/iRD1+CEP250L-1 4h aux DAPI panel-2.jpg]

-IAA

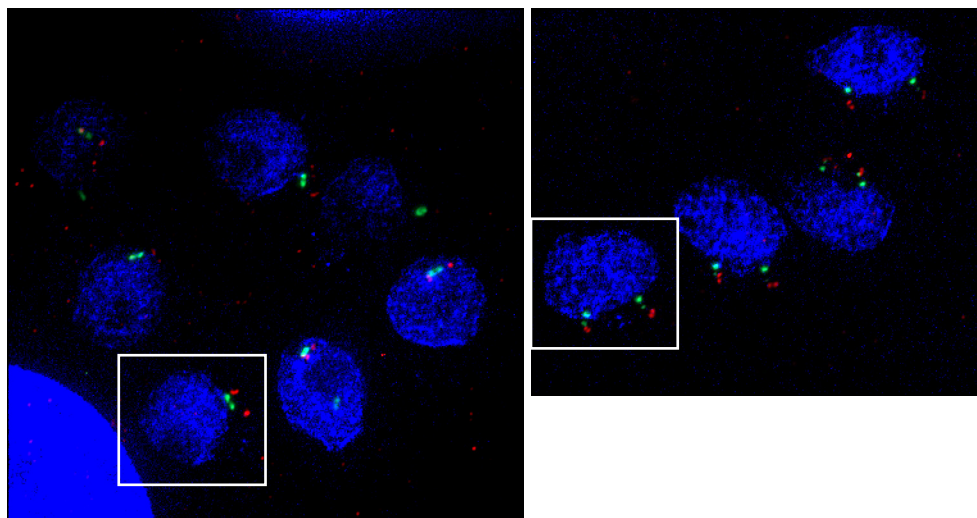

+IAA

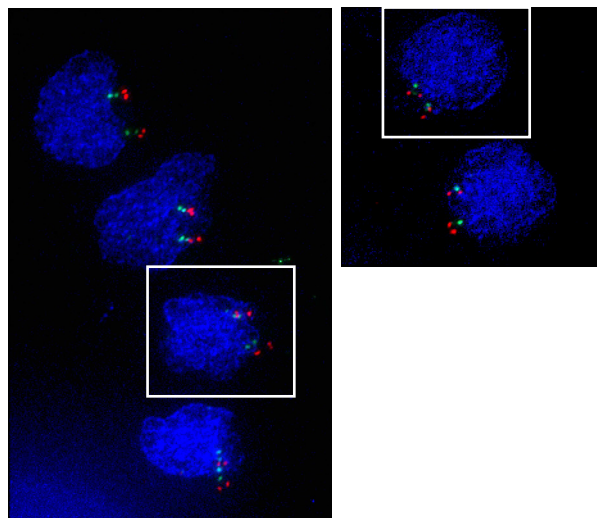

Supplement: Supplementary file 14 — Source data Fig. 7 [file 44318_2024_95_MOESM14_ESM.zip › SD Figure 7/7F/readme.pdf]

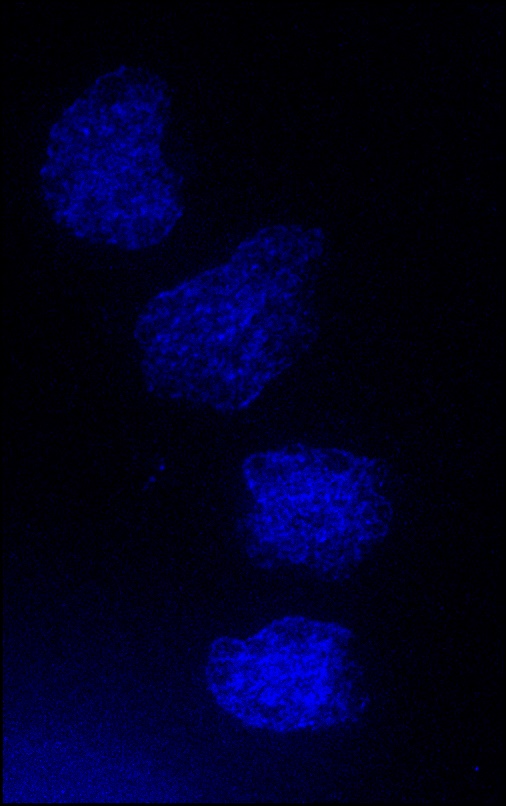

Supplement: Supplementary file 14 — Source data Fig. 7 [file 44318_2024_95_MOESM14_ESM.zip › SD Figure 7/7F/iRD1+CEP250L-1 4h aux DAPI panel-1.jpg]

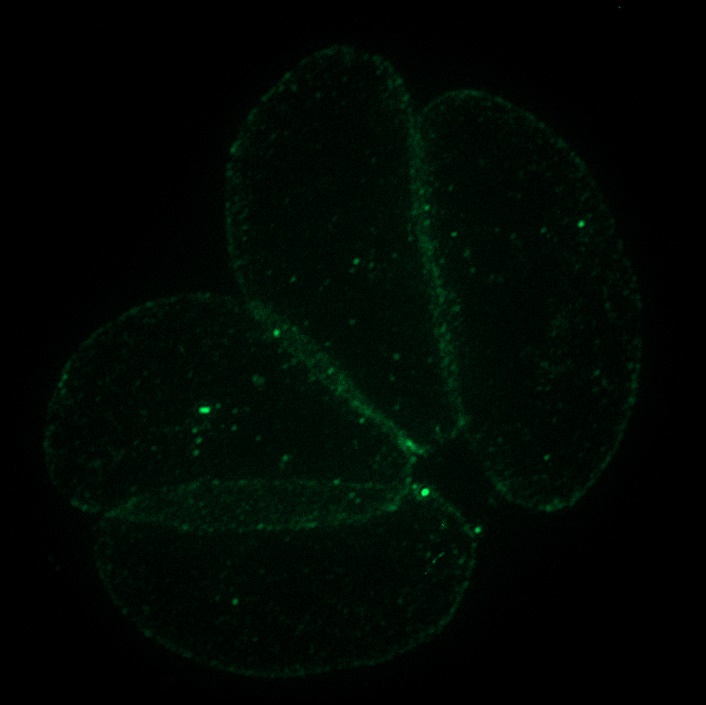

Supplement: Supplementary file 14 — Source data Fig. 7 [file 44318_2024_95_MOESM14_ESM.zip › SD Figure 7/7H/iRD1+CEP250L-1 4h aux IMC1.jpg]

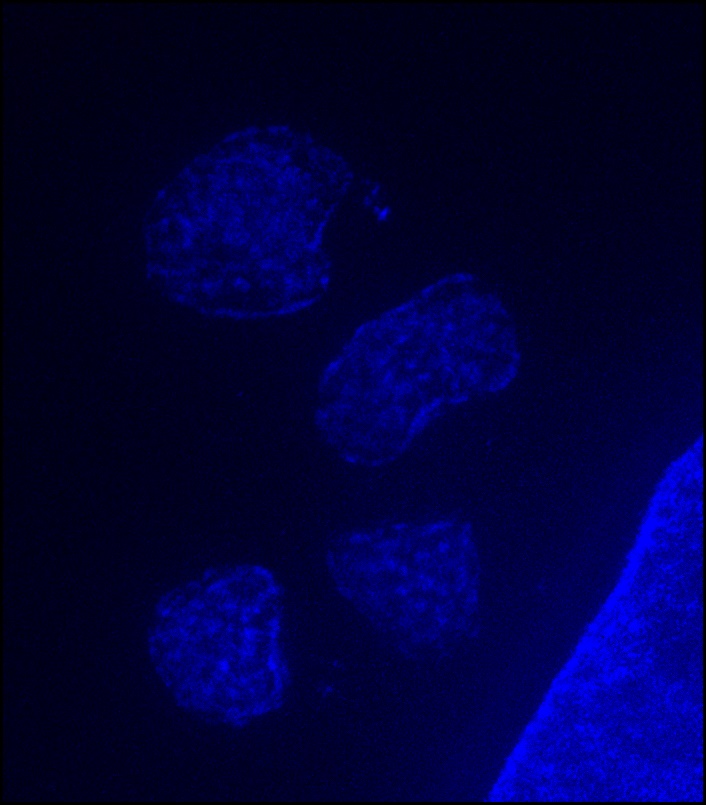

Supplement: Supplementary file 14 — Source data Fig. 7 [file 44318_2024_95_MOESM14_ESM.zip › SD Figure 7/7H/iRD1+CEP250L-1 -aux DAPI.jpg]

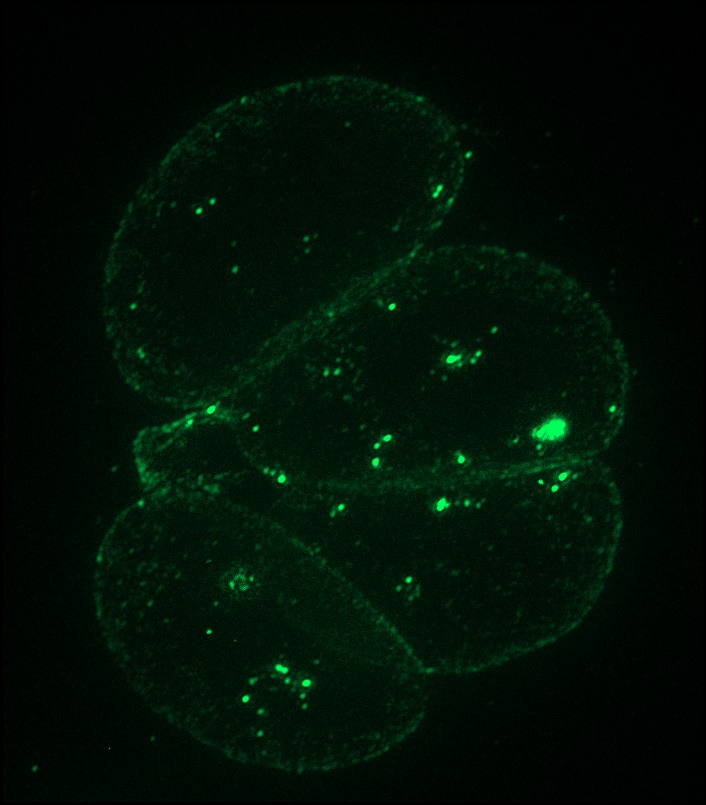

Supplement: Supplementary file 14 — Source data Fig. 7 [file 44318_2024_95_MOESM14_ESM.zip › SD Figure 7/7H/iRD1+CEP250L-1 -aux IMC1.jpg]

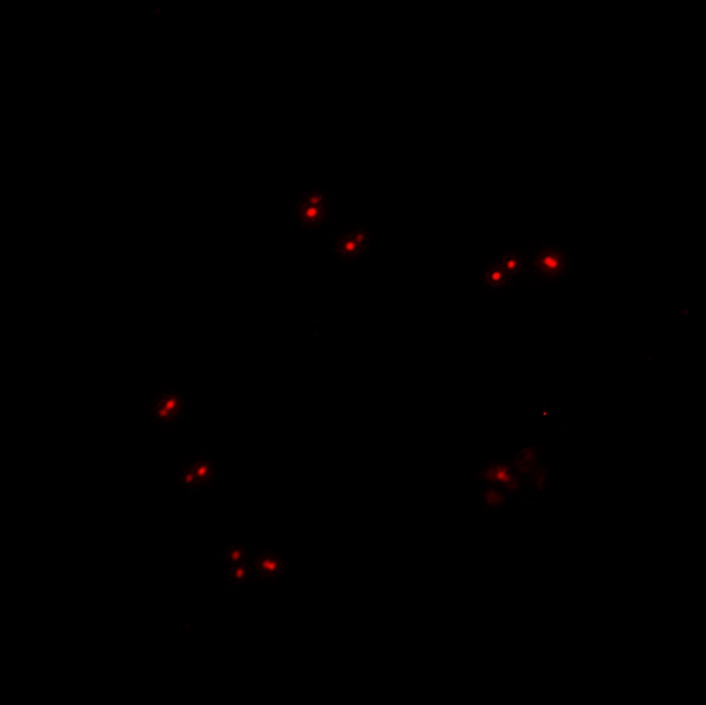

Supplement: Supplementary file 14 — Source data Fig. 7 [file 44318_2024_95_MOESM14_ESM.zip › SD Figure 7/7H/iRD1+CEP250L-1 4h aux myc.jpg]

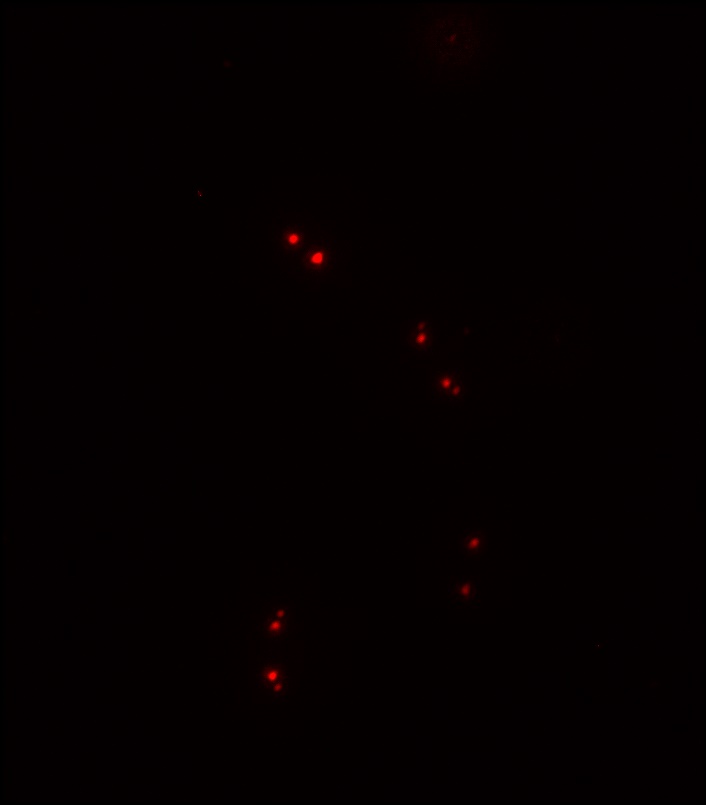

Supplement: Supplementary file 14 — Source data Fig. 7 [file 44318_2024_95_MOESM14_ESM.zip › SD Figure 7/7H/iRD1+CEP250L-1 -aux myc.jpg]

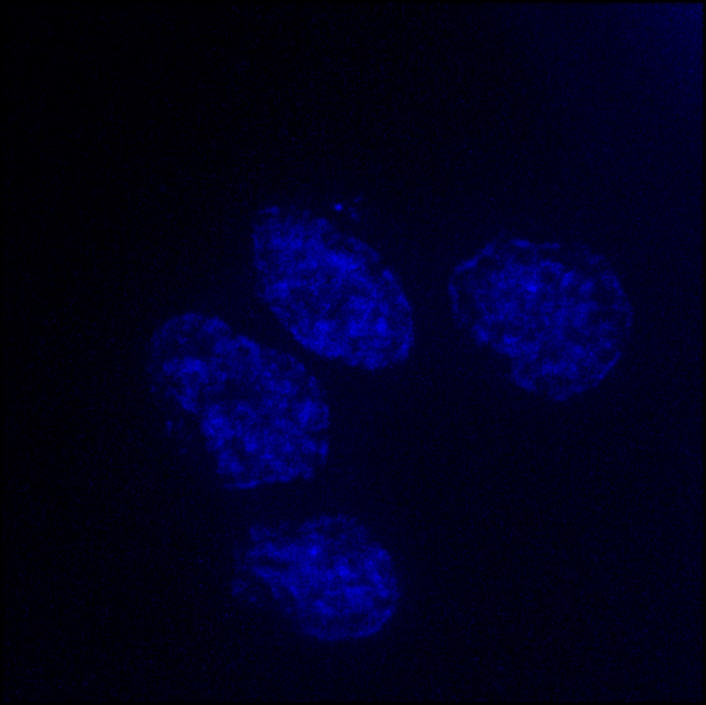

Supplement: Supplementary file 14 — Source data Fig. 7 [file 44318_2024_95_MOESM14_ESM.zip › SD Figure 7/7H/iRD1+CEP250L-1 4h aux DAPI.jpg]
