# Supplementary material for: The Crk4-Cyc4 complex regulates G2/M transition in Toxoplasma gondii
Source: EMBO J. 2024 Apr 10;43(11):2094–126. doi: 10.1038/s44318-024-00095-4 (PMC11148040; doi:10.1038/s44318-024-00095-4)
Supplement: Supplementary file 16 — Expanded View Figures [file 44318_2024_95_MOESM16_ESM.pdf]

Expanded View Figures

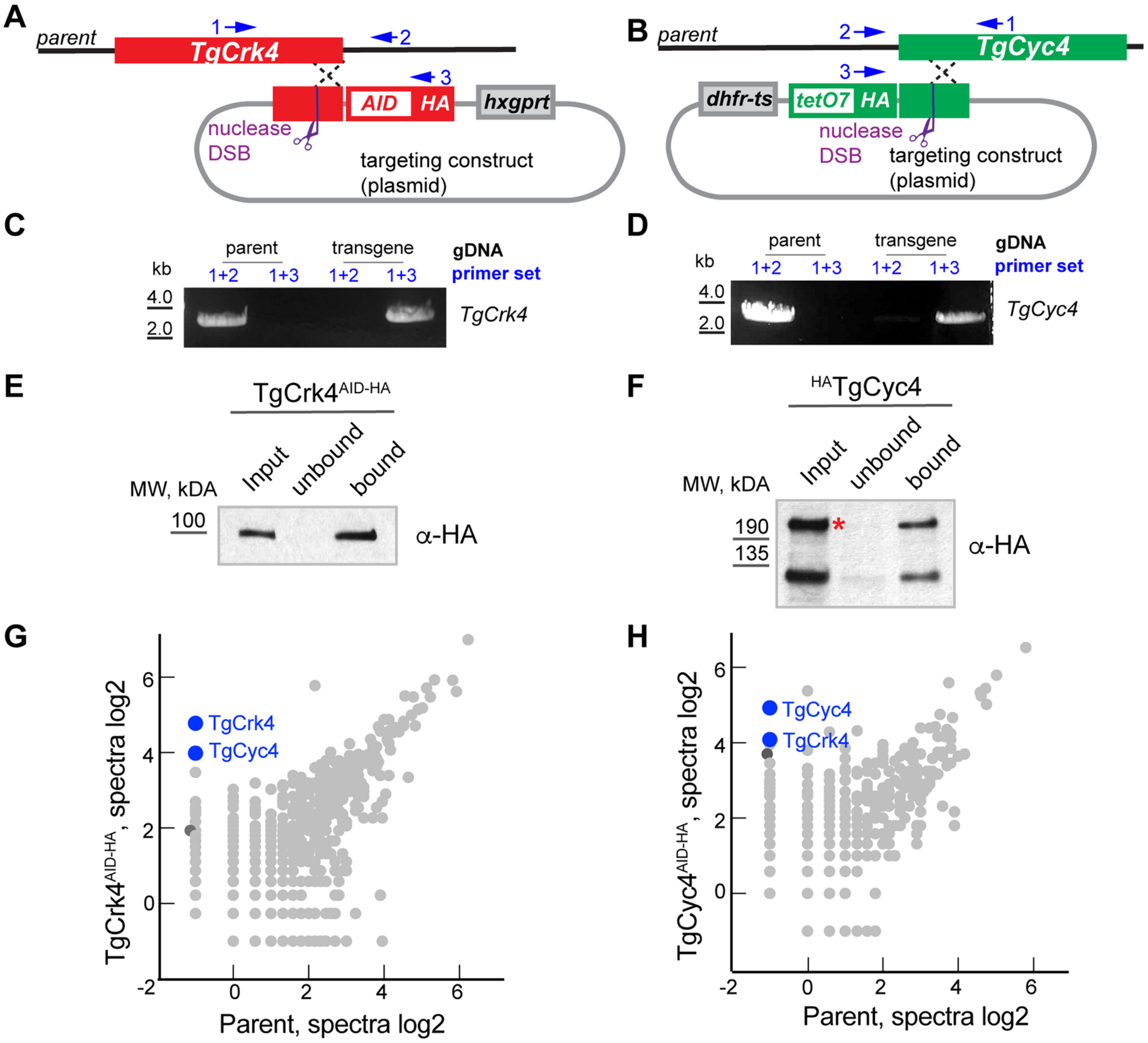

Figure EV1. Generation and analysis of conditional expression models for TgCrk4 and TgCyc4.

(**A**) Schematics for constructing TgCrk4 AID-modified gene. Targeting plasmid included 3′ fragment of *TgCrk4* genomic locus fused with encoded sequence for mini-version of AID (mAID), 3xHA (HA) epitopes and the drug-selection marker *hxgprt* gene (gray box). The plasmid linearization with a unique endonuclease induced recombination at the *TgCrk4* locus. Schematics also indicates the relative position of the primers used to conform the TgCrk4 knock-in (**C**). (**B**) Schematics for constructing TgCyc4 Tet-OFF-modified gene. The 5′ fragment of *TgCyc4* genomic locus was amplified and cloned into the targeted plasmid to create N-terminal fusion with 3xHA (HA) epitopes (gray box). The plasmid linearization with a unique endonuclease induced recombination at the *TgCyc4* locus. Schematics also indicates the relative position of the primers used to conform the TgCyc4 knock-in (**D**). (**C**, **D**) PCR analysis of the parental and transgenic lines expressing $TgCrk4^{AID\text{-}HA}$ or $^{HA}TgCyc4$. The combination of the primers used to detect either native or recombined locus are shown. (**E**, **F**) Western blot analysis of TgCrk4 (**E**) and TgCyc4 (**F**) immunoprecipitation. The protein complexes were immunoisolated from the soluble fraction [In] (input) of parasites co-expressing endogenous $TgCrk4^{AID\text{-}HA}$ or $^{HA}TgCyc4$. Beads with precipitated complexes (**B**) and depleted soluble fraction [Un] (unbound) were probed with α-HA (α-rat IgG-HRP) antibodies to confirm efficient pulldown of the target proteins. Red asterisk marks the full-length TgCyc4 protein. (**G**, **H**) The results of the SAINT analysis of TgCrk4 (**G**) and TgCyc4 (**H**) proteomes. The log2 values of the protein spectra detected by mass-spectrometry analysis of the parent parasites and parasites expressing $TgCrk4^{AID\text{-}HA}$ or $^{HA}TgCyc4$ are plotted on the graph. The TgCrk4-TgCyc4 complex is indicated with blue color. TGME49_247040 protein is shown as a dark gray circle.

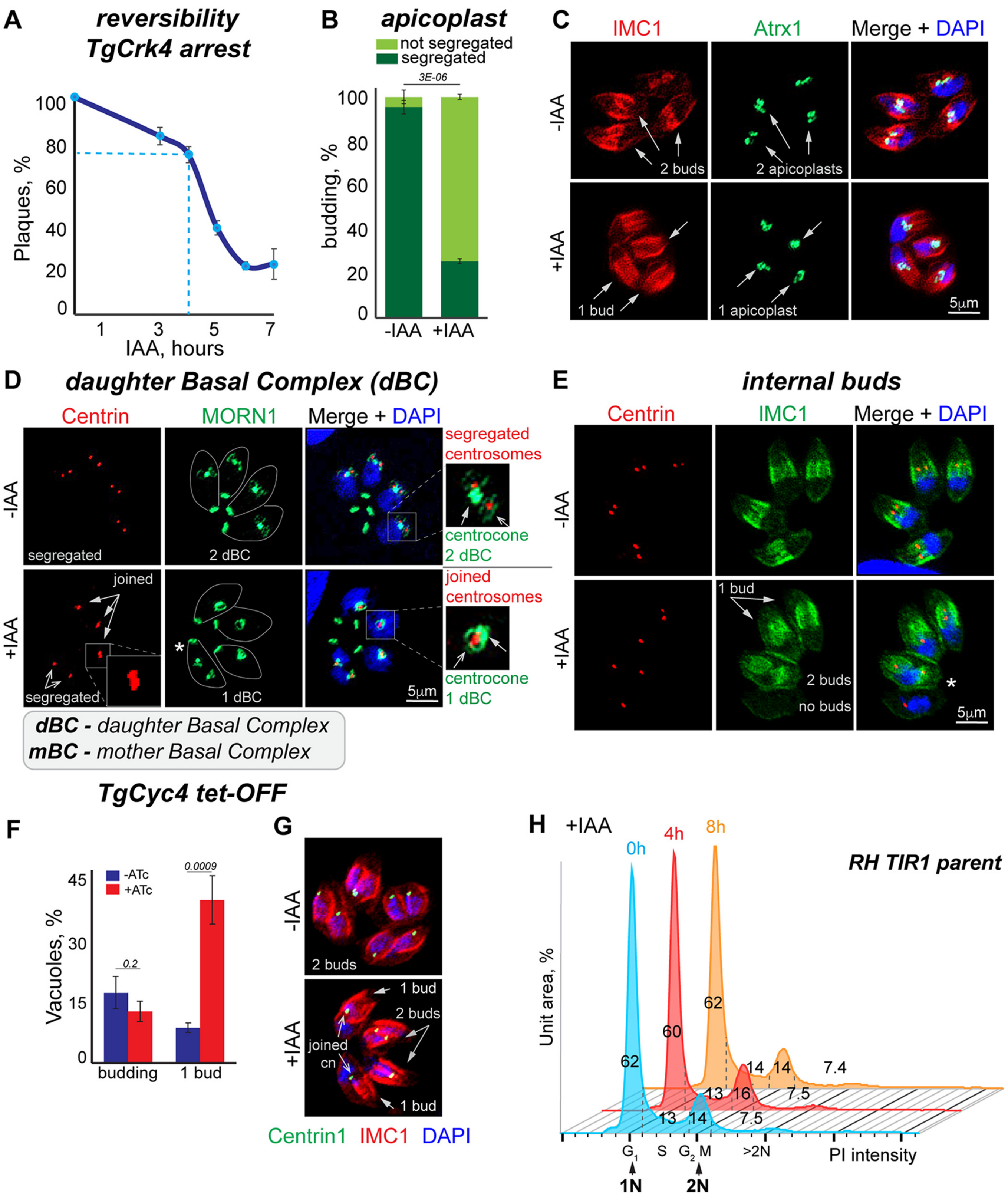
A
reversibility TgCrk4 arrest
Plaques, %
IAA, hours
B
apicoplast
not segregated
segregated
3E-06
budding, %
-IAA +IAA
C
IMC1
Atrx1
Merge + DAPI
-IAA
+IAA
2 buds
2 apicoplasts
1 bud
1 apicoplast
5µm
D
daughter Basal Complex (dBC)
Centrin
MORN1
Merge + DAPI
segregated centrosomes
segregated
2 dBC
centrocone
2 dBC
joined
joined centrosomes
1 dBC
centrocone
1 dBC
dBC - daughter Basal Complex
mBC - mother Basal Complex
E
internal buds
Centrin
IMC1
Merge + DAPI
1 bud
2 buds
no buds
5µm
TgCyc4 tet-OFF
F
-ATc
+ATc
0.0009
0.2
Vacuoles, %
budding
1 bud
G
2 buds
1 bud
joined cn
Centrin1 IMC1 DAPI
H
+IAA
0h 4h 8h
RH TIR1 parent
Unit area, %
PI intensity
G1 S G2 M >2N
1N 2N

 © The Author(s)

Figure EV2. Characterization of TgCrk4 knockdown phenotype.

(**A**) The reversibility of the TgCrk4-induced block was determined by plaque assay. Freshly invaded RH TgCrk4^{AID-HA} parasites were incubated with 500µM IAA for indicated times before the medium was replaced with normal growth medium without IAA to allow for plaque development. The plotted mean −/+ SD values represent the average plaque numbers from three independent measurements. (**B**) Quantification of the apicoplast segregation defect caused by RH TgCrk4^{AID-HA} deficiency. The number of the vacuoles containing budding parasites that segregated or did not segregate apicoplast were quantified in non-treated and treated with 500 µM IAA for 8 h parasites using TgAtrx1 staining as shown on (**C**). The longer IAA treatment allowed development of the bigger buds to aid quantifications. A hundred vacuoles of the budding parasites were examined in three independent experiments. The mean −/+ SD values are plotted on the graph. The unpaired two-sided *t* test value is shown. (**C–E**) Immunofluorescent microscopy analysis of RHΔ*Ku80TIR1* TgCrk4^{AID-HA} tachyzoites incubated without (−IAA) or with 500 µM IAA for 4 h (+IAA). (**C**) Parasites were co-stained with α-TgAtrx1 (α-mouse IgG Fluor 488), α-TgIMC1 (α-rabbit IgG Fluor 488) antibodies and DAPI to visualize apicoplast, internal buds and nuclei. (**D**) Parasites were co-stained with α-TgMORN1 (α-rabbit IgG Fluor 488), α-Centrin1 (α-mouse IgG Fluor 488) antibodies and DAPI to determine the number of centrosomes and daughter basal complexes (dBC). (**E**) Parasites were co-stained with α-TgIMC1 (α-rabbit IgG Fluor 488), α-Centrin1 (α-mouse IgG Fluor 488) antibodies and DAPI to visualize internal buds and determine the number of centrosomes per parasite. White asterisk indicates normally dividing tachyzoite. (**F**) Quantification of the defects caused by TgCyc4 downregulation. The TgCyc4 Tet-OFF parasites were grown without or with ATc for 16 h. The mean −/+ SD number of the vacuoles containing budding parasites and parasites forming a single bud in three independent experiments is plotted on the graph. The unpaired two-sided *t* test values are shown. (**G**) The immunofluorescence microscopy analysis of the TgCyc4 Tet-OFF parasites grown without or with ATc for 16 h. The parasites were co-stained with α-Centrin1 (α-mouse IgG Fluor 488), α-TgIMC1 (α-rabbit IgG Fluor 488) antibodies and DAPI to visualize centrosomes, internal buds and nuclei. The quantifications of three independent experiments are shown on (**F**). (**H**) FACScan analysis of DNA content of the parental RHΔ*Ku80TIR1* strain non-treated (0 h, IAA, blue plot) or treated with auxin for 4 h (red plot) and 8 h (orange plot). The results of one of three independent experiments are shown. Dashed lines show the gates used to segregate parasites containing non-replicated (G_1: $<1-1N$), replicating (S: 1–<2N), replicated (G_2+M: 2N) and over-replicated (>2N) DNA.

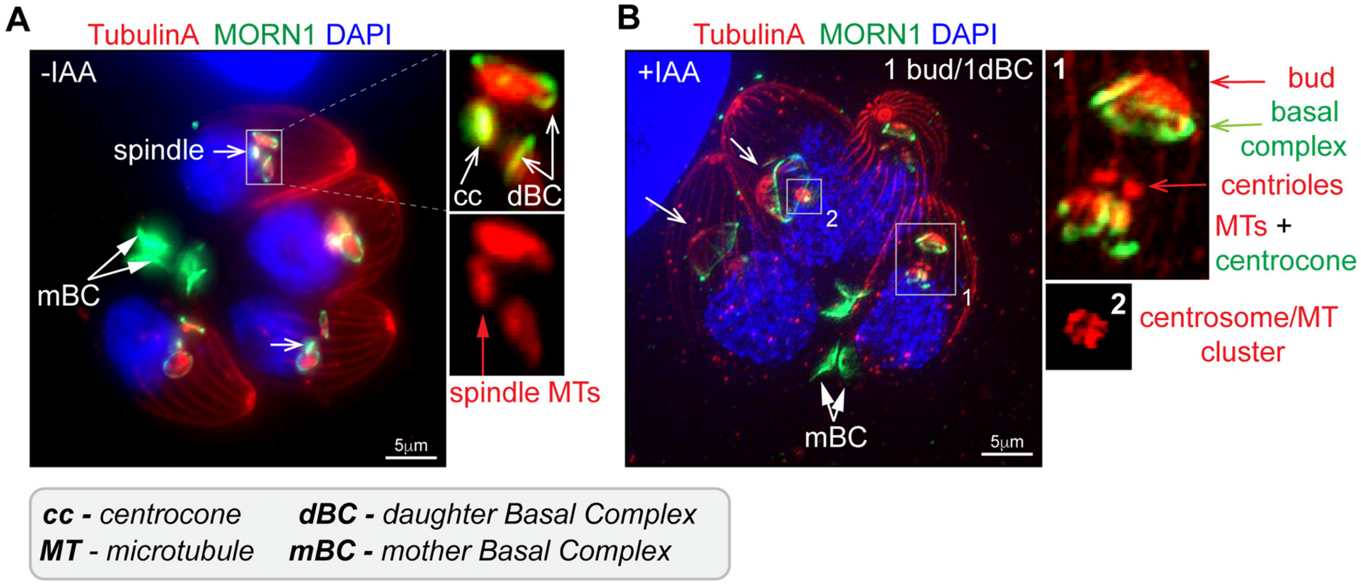

Figure EV3. Microscopy analysis of TgCrk4-deficient tachyzoites.

(**A**, **B**) The ultra-expansion microscopy analysis of RHΔ*Ku80TIR1* TgCrk4$^{AID\text{-}HA}$ expressing (**A**) and deficient (**B**) tachyzoites. (**A**) Co-staining of Tubulin A (α-TubulinA/α-mouse IgG Fluor 568) and TgMORN1 (α-MORN1/α-rabbit IgG Flour 488) shows spindle microtubules located in the extended centrocone and two daughter basal complexes (dBC) encircling subpellicular microtubules of the daughter bud. Nucleus stained with DAPI (blue). (**B**) Co-staining of the TgCrk4-deficient parasites (4 h, 500 μM IAA) shows assembly of a single daughter bud.

 © The Author(s)

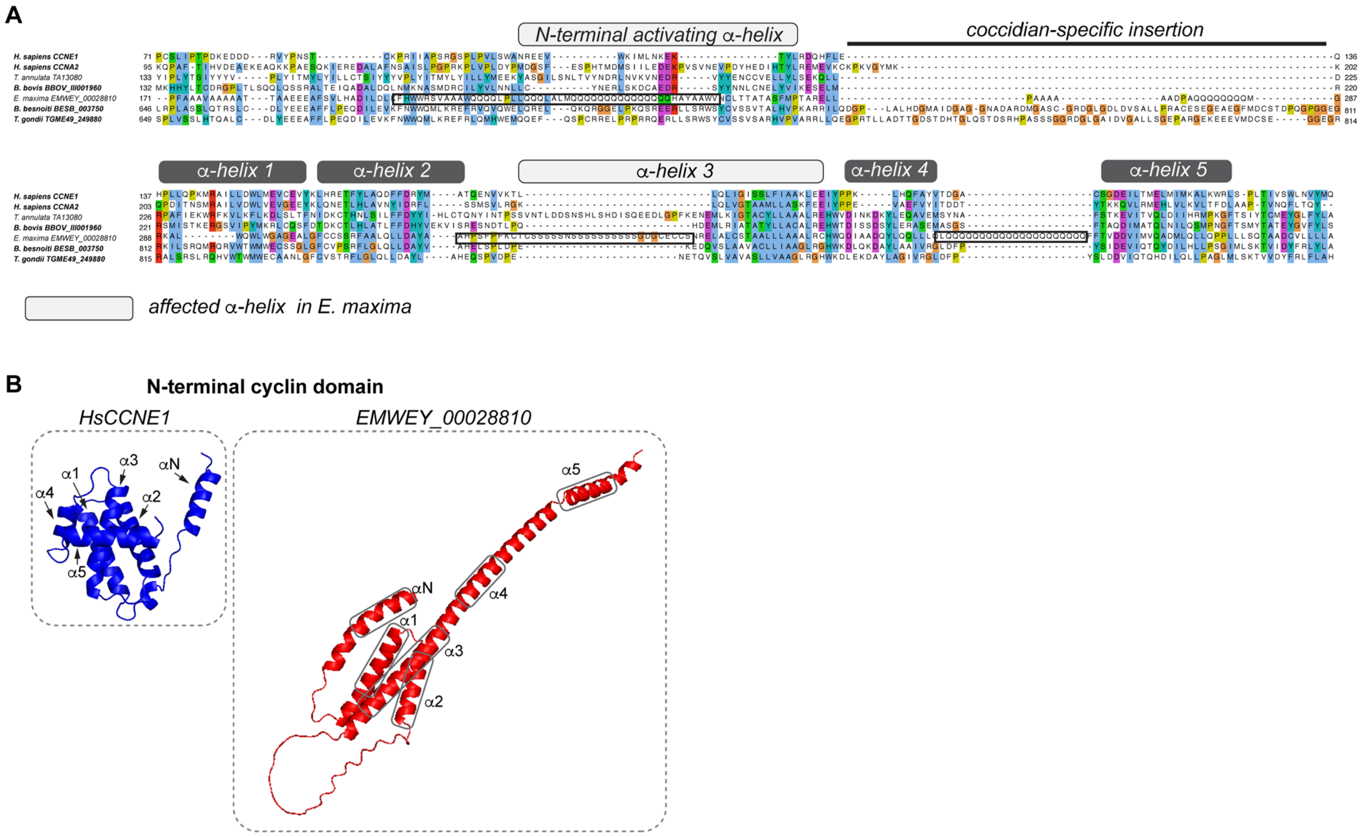

Figure EV4. Structural analysis of TgCyc4-related proteins.

(**A**) Alignment of the N-terminal cyclin domains of apicomplexan Cyc4 proteins with *H. sapiens* Cyclin A2 and Cyclin E1 (MUSCLE). (**B**) Predicted folding of the cyclin domains of *H. sapiens* Cyclin E1 and Eimeria Cyc4.

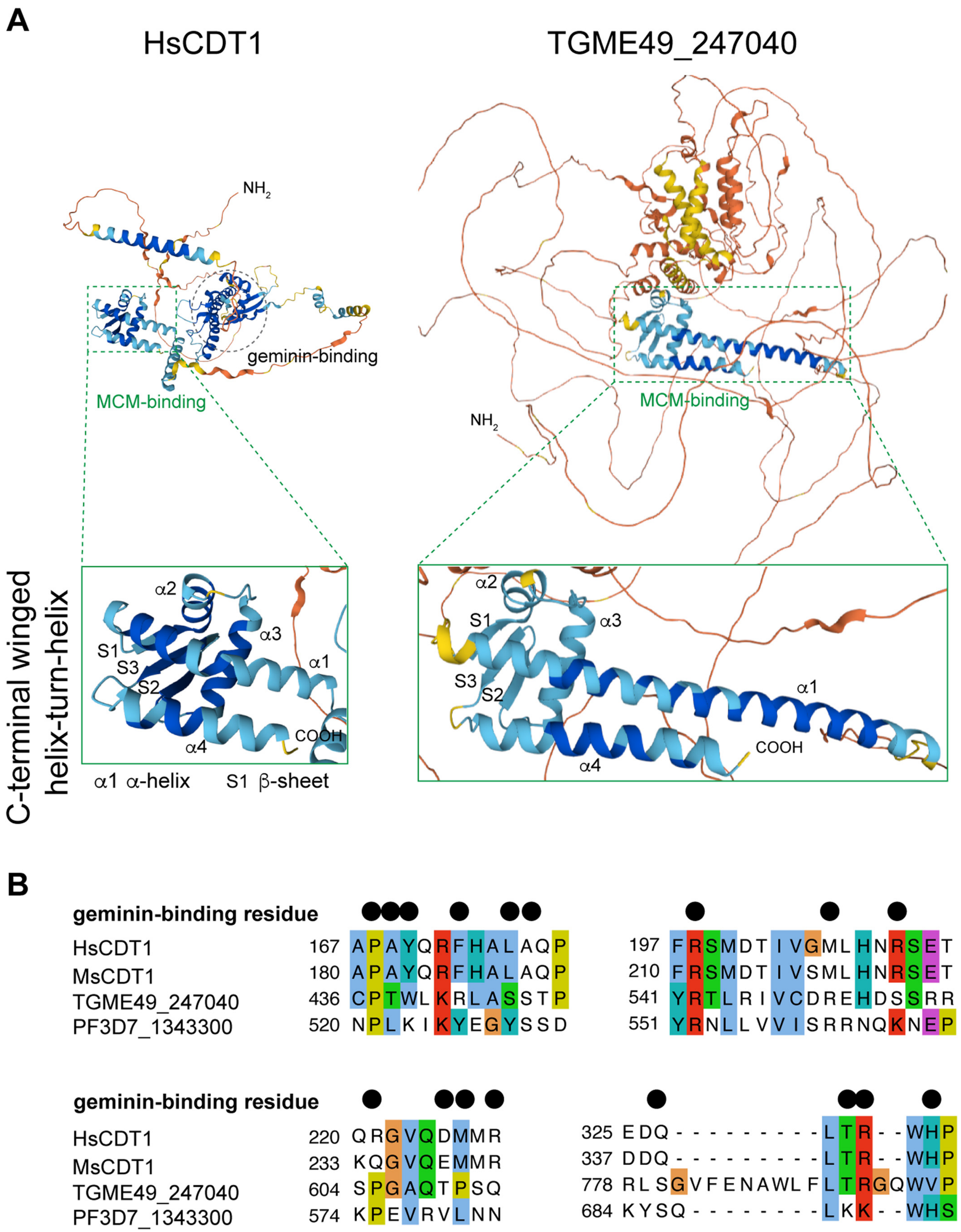
A
HsCDT1
TGME49_247040
NH2
geminin-binding
MCM-binding
NH2
MCM-binding
C-terminal winged helix-turn-helix
α2
α3
S1
S3
S2
α1
α4
COOH
α1 α-helix
S1 β-sheet
α2
S1
α3
S3
S2
α1
α4
COOH
B
geminin-binding residue
HsCDT1 167 APAYQRFHALAQP 197 FRSMDTIVGMLHNRSET
MsCDT1 180 APAYQRFHALAQP 210 FRSMDTIVSMLHNRSET
TGME49_247040 436 CPTWLKRLASSTP 541 YRTLRIVCDREHDSSRR
PF3D7_1343300 520 NPLKIKYEGYSSD 551 YRNLLVVISRRNQKNEP
geminin-binding residue
HsCDT1 220 QRGVQDMMR 325 EDQ---------LTR--WHP
MsCDT1 233 KQGVQEMMR 337 DDQ---------LTR--WHP
TGME49_247040 604 SPGAQTPSQ 778 RLSGVFENAWLFLTRGQWVP
PF3D7_1343300 574 KPEVRVLNN 684 KYSQ--------LKK--WHS

Figure EV5. Structural analysis of *Toxoplasma* CDT1-related protein.

(**A**) Folding prediction of *H. sapiens* CDT1 and *T. gondii* TGME49_247040 factor (AlphaFold2). The enlarged regions show C-terminal winged helix-turn-helix structure responsible for interaction with MCM complex. (**B**) Alignment of the central domain of *H. sapiens* CDT1 and *M. musculus* CDT1 with corresponding regions of *T. gondii* TGME49_ 247040 and *P. falciparum* PF3D7_1343300. The black dot indicates the residues involved in CDT1 interaction with inhibitor Geminin. Note a poor conservation of the Geminin-binding sites in apicomplexans.

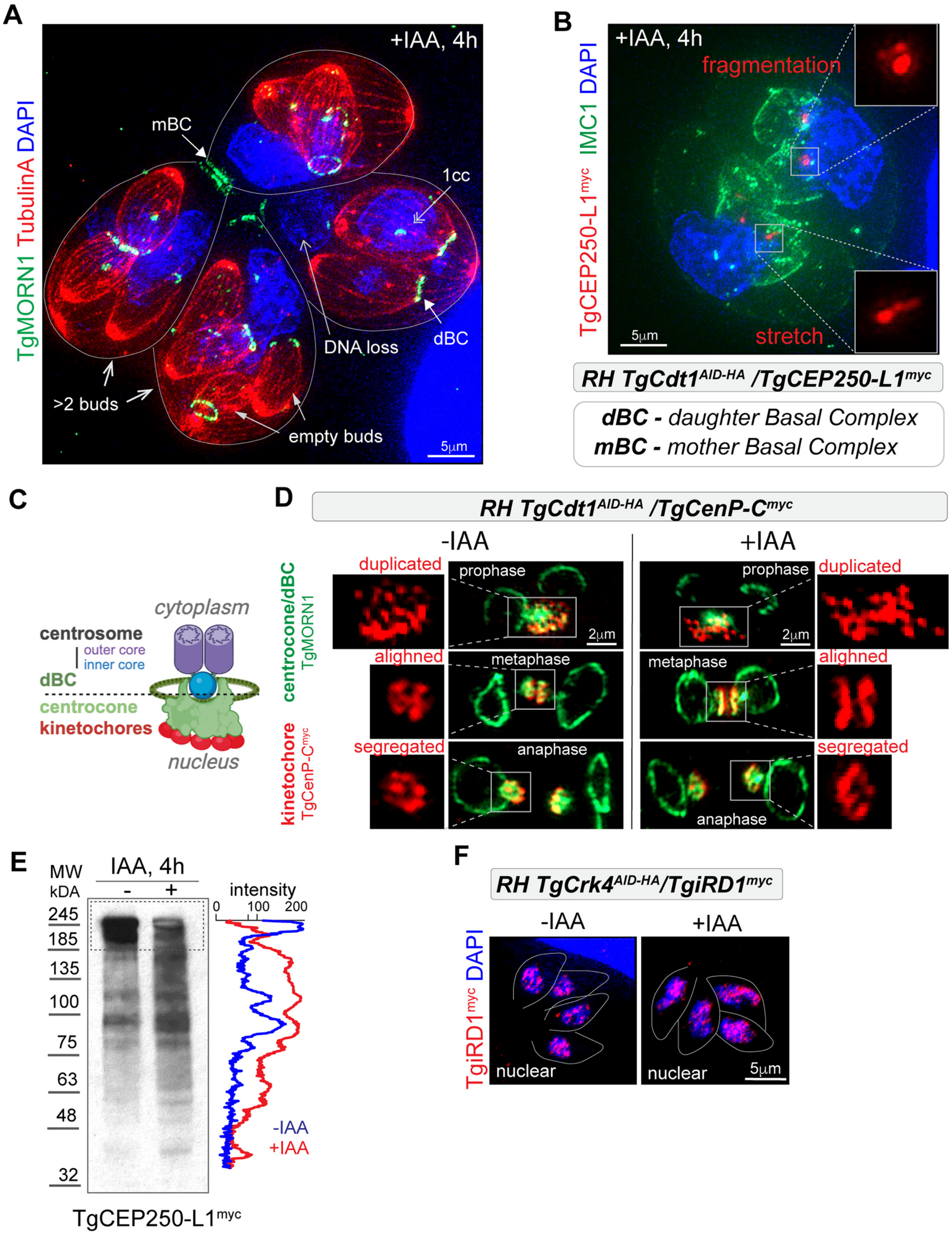
A
+IAA, 4h
TgMORN1 TubulinA DAPI
mBC
1cc
DNA loss
dBC
>2 buds
empty buds
5μm
B
+IAA, 4h
fragmentation
stretch
TgCEP250-L1myc IMC1 DAPI
5μm
RH TgCdt1AID-HA /TgCEP250-L1myc
dBC - daughter Basal Complex
mBC - mother Basal Complex
C
cytoplasm
centrosome
outer core
inner core
dBC
centrocone
kinetochores
nucleus
D
RH TgCdt1AID-HA /TgCenP-Cmyc
-IAA
+IAA
centrocone/dBC TgMORN1
kinetochore TgCenP-Cmyc
duplicated
prophase
2μm
aligned
metaphase
segregated
anaphase
E
MW kDA
IAA, 4h
- +
245
185
135
100
75
63
48
32
intensity
0 100 200
-IAA
+IAA
TgCEP250-L1myc
F
RH TgCrk4AID-HA/TgiRD1myc
-IAA
+IAA
TgiRD1myc DAPI
nuclear
5μm

 © The Author(s)

Figure EV6. The phenotypic analysis of the TgiRD1 deficient tachyzoites.

(**A**) Ultra-expansion microscopy analysis of RHΔ*Ku80TIR1* TgiRD1^{AID-HA} deficient tachyzoites. Staining with Tubulin A (α-TubulinA/α-mouse IgG Fluor 568) visualizes the mother and the daughters' subpellicular microtubules (buds). The TgMORN1 (α-MORN1/α-rabbit IgG Flour 488) staining shows changes to centrocone and segregates the mother (mBC) and the daughter basal complexes (dBC). DNA mis-segregation defect is depicted with nuclear DAPI stain (blue). (**B**) The ultra-expansion microscopy images of RHΔ*Ku80TIR1* TgiRD1^{AID-HA} deficient tachyzoites expressing TgCEP250-L1^{myc}. The inner core of the centrosome was detected with α-myc (α-rabbit IgG Flour 568) antibodies, the parasite surface with α-IMC1 (α-mouse IgG Fluor 488) and nucleus with DAPI stain. The inner core changes caused by TgiRD1 depletion (+IAA, 4 h) are highlighted in the insets. (**C**) Schematics of the *T. gondii* perinuclear structures including the bipartite centrosome, centrocone and kinetochores. The drawing depicts one half of the mitotic figure. The dotted line separates nucleoplasm and cytoplasm. dBC daughter basal complex. (**D**) The ultra-expansion microscopy images of RHΔ*Ku80TIR1* TgiRD1^{AID-HA} tachyzoites expressing TgCenP-C^{myc} after 4 h incubation without or with IAA. Three stages of mitosis are shown. To determine the relative position of kinetochores and centrocone, the samples were co-stained with α-myc (α-mouse IgG Flour 568) antibodies and α-TgMORN1 (α-rabbit IgG Fluor 488). (**E**) Western blot analysis of the TgCEP250-L1^{myc} expression in the TgiRD1 expressing (−IAA) and deficient (+IAA, 4 h) parasites probed with α-myc. An overexposed image of TgCEP250-L1^{myc} (Fig. 7G) shows accumulation of the degradation products. The densitometry analysis of the image is shown on the right (ImageJ). (**F**) IFA analysis of TgiRD1^{myc} localization in RHΔ*Ku80TIR1* parasites expressing (-IAA) or deficient for TgCrk4^{AID-HA} (+ IAA, 4 h). Nuclear localization of TgiRD1^{myc} was detected with α-myc (α-rabbit IgG Fluor 568) antibodies and DAPI stain (blue).

© The Author(s)
